# A bespoke non-invasive approach to predict fetal genotype in pregnancy

**DOI:** 10.3389/fmed.2026.1745404

**Published:** 2026-09-28

**Authors:** Carol A. Wang, Carlos Riveros, John R. Attia, Rodney J. Scott, Craig E. Pennell

**Affiliations:** 1 School of Medicine and Public Health, University of Newcastle, Newcastle, NSW, Australia; 2 Mothers' and Babies' Research Centre, Hunter Medical Research Institute, Newcastle, NSW, Australia; 3 School of Biomedical Sciences and Pharmacy, University of Newcastle, Newcastle, NSW, Australia; 4 Division of Molecular Medicine, Pathology North, John Hunter Hospital, Newcastle, NSW, Australia; 5 Department of Maternity and Gynecology, John Hunter Hospital, Newcastle, NSW, Australia

**Keywords:** cell-free DNA, fetal growth, fetal SNP, NIPT, non-invasive prenatal testing, SNP-array genotyping

## Abstract

**Introduction:**

The fetal genome influences fetal growth; however, it is currently not used to customize fetal growth charts due to the need for invasive testing. Total cell-free-DNA (T-cfDNA) from maternal blood during pregnancy contains maternal-cfDNA and fetal-cfDNA. T-cfDNA offers the opportunity to non-invasively determine fetal single nucleotide polymorphisms (SNPs). This study aimed to determine the feasibility of genotyping fetal SNPs in pregnancy using parental genomic-DNA (gDNA) and maternal T-cfDNA, and computing fetal polygenic scores (PGS) as proxies for several health domains.

**Methods:**

Parental and cord blood gDNA, and maternal T-cfDNA were collected. Genotyping was performed using Illumina Global Screening Array. Signal intensity parameters (SIPs) were extracted for genotyped SNPs. Fetal SNPs were predicted using corresponding paired parental SNPs when both parents were homozygous, and from maternal gDNA SIP confidence intervals otherwise. Six PGSs for phenotypes including birthweight-PGS were computed using predicted fetal SNPs. Predictions were verified against genotyped cord blood.

**Results:**

The average fetal SNP prediction accuracy was 86.39% (95% CI: 85.94-86.84%). Improved performance was observed when computing PGSs with a greater number of SNPs; coefficient of variation ranged from 5.6-16.5%.

**Discussion:**

Results suggested moderate predictive performance. Improvements in the accuracy of SNP prediction are needed before clinical implementation. This may be through advanced bioinformatics methods or genotyping methodology.

## 1 Introduction

The discovery of cell-free fetal DNA (cffDNA) in total cell-free DNA (T-cfDNA) obtained from maternal blood during pregnancy marked an important milestone for non-invasive prenatal testing (NIPT). Several national governing bodies including the American College of Obstetricians and Gynecologists (ACOG), the UK National Screening Committee (UK NSC), the Royal Australian and New Zealand College of Obstetricians and Gynecologists (RANZCOG), and Royal Australian College of General Practitioners (RACGP) are increasingly offering NIPT for pregnant women as an option as part of prenatal screening for fetal chromosomal anormalies (1–4). NIPT of T-cfDNA from pregnant women provides insight into the fetal genetics during pregnancy. Currently, it is used primarily to screen for aneuploidy and microdeletions (1, 2, 5). In targeted populations, NIPT is also used to screen for specific monogenic diseases (6–8). Conventional invasive testing methods allow direct access to fetal DNA; however, these techniques are associated with complications including amniotic fluid leakage, chorioamnionitis, and miscarriage (9). The implementation of invasive testing as part of routine care to identify associations with common health outcomes is not ethically justifiable at a population level. Whole-genome sequencing (WGS) made it possible to sequence the human genome. The financial implications and computational demands, however, prohibit access for the general population and the healthcare system. Further, WGS is more suited for studying rare hereditary diseases as opposed to common health outcomes which impact a larger proportion of the general population.

Birthweight is a common phenotype studied in a pregnant population. The genetic influence on birthweight has been established since the 1950s (10). Recent twin and family studies indicate that maternal and fetal common single-nucleotide-polymorphisms (SNPs) could explain up to 69% of the variability in birthweight, with maternal and fetal genetics contributing between 3%–22% and 24%–69%, respectively (11–15). In a series of studies investigating SNPs and birthweight, Horikoshi et al. presented compelling evidence that common SNPs explained approximately 15% of the variability in birthweight, an effect size of similar magnitude to the effects of maternal smoking during pregnancy and maternal BMI (11, 16). The most extensive genome-wide-association-study (GWAS) analysis of birthweight performed in the Early Growth Genetics (EGG) Consortium and UK Biobank (UKBB) which included up to 321,223 individuals identified 146 fetal SNPs and 72 maternal SNPs that influenced fetal growth (17). Of note, several of these common SNPs identified also demonstrated associations with adverse childhood or adult cardiometabolic outcomes (11, 18–20).

Current resource and knowledge gaps have hindered examining fetal SNPs in pregnancy in a practical, inexpensive, and non-invasive manner. SNP-arrays can capture >500 K common SNPs in a cost-effective manner and are less computationally demanding than WGS. SNP-arrays are commonly used to examine associations between genetic variants and common health outcomes. Further, most common diseases and traits are multifactorial and polygenic in nature (21). Polygenic scores (PGSs), developed from common SNPs, could provide insights into the genetic pre-dispositions and compounded impact on the targeted phenotypes investigated. The use of SNP-arrays to analyze T-cfDNA from maternal circulation to gain insight into the fetus' genetics could offer an economically affordability and accessibility solution suited for the general population and the healthcare system.

There are several challenges when analyzing T-cfDNA from maternal circulation. Fetal fraction (FF), the proportion of T-cfDNA derived from the fetal genome, is critical when interpreting results (22). Specifically, low FF reduces accuracy; high FF, on the other hand, is a marker for potential pregnancy complication (22). FF typically ranges between 10% and 15% of T-cfDNA at 10–20 weeks' gestation (23, 24); however, limitations exist within current laboratory and bioinformatics pipelines to allow sufficiently high accuracy in the determination of individual fetal SNP genotypes from standalone genotyping of maternal T-cfDNA. As a result of these limitations, the ability to determine fetal genotype for multiple SNPs in a safe, non-invasive manner during pregnancy remains a challenge.

This study aims to determine the feasibility of developing a safe and non-invasive method to derive fetal genotype in pregnancy using SNP-array, and to determine its potential to develop various PGSs, including birthweight-PGS, that have the potential to customize the assessment of fetal growth.

## 2 Materials and methods

### 2.1 Study population and sample collection

Study participants were recruited through the Fetal Prediction Study (FPS). A total of 30 families with intent for planned caesarean section delivery at the John Hunter Hospital (JHH) were recruited when they attended their 36-week gestation antenatal clinic appointment. Paternal blood, in addition to maternal blood, was collected at the time of recruitment if the baby's biological father was also present. Cord blood was collected at the birth of the baby, and missing paternal blood was collected at this opportunity, if possible.

Ethics approvals were granted by the Hunter New England Human Research Ethics Committee (HREC Ethics Approval 2020ETH01409) on 30 June 2020, before study commencement. All study families provided written consent to participate in the study before recruitment.

### 2.2 Laboratory methods

Maternal blood was collected in Streck Cell-Free DNA Blood Collection Tubes®, while paternal and cord blood were collected in tubes containing EDTA. Genomic DNA was extracted from parental and cord blood. T-cfDNA, a mixture of maternal cfDNA and fetal cfDNA, was extracted from maternal blood. Samples were processed within two hours from collection to minimize the potential for sample degradation. Briefly, the blood samples were centrifuged to give plasma, buffy coat and erythrocytes aliquots. Plasma and buffy coat aliquots were then stored appropriately till the end of the recruitment phase for the batch extraction of genomic and T-cfDNA from buffy coat and plasma aliquots, respectively. Genomic DNA was isolated from maternal, paternal, and cord blood buffy coat aliquots using the Qiagen DNeasy blood and tissue kit according to published protocols (25), while T-cfDNA was isolated from maternal plasma aliquots using the QIAGEN QIAamp Circulating Nucleic Acid Kit according to published protocols (26). All gDNA and T-cfDNA samples were quantified after isolation. All gDNA was genotyped using Illumina Infinium Global Screening Array-24 v3.0 (GSA) Beadchip as per guidelines in the Illumina Infinium HTS Assay Reference Guide (27). Compared to gDNA, T-cfDNA is not present in abundance in the circulation. In this study, only T-cfDNA with yields greater than 25 ng were genotyped.

### 2.3 Analytic method

Variant calling was performed using the Illumina Array Analysis Platform Genotyping Command Line Interface (iaap-cli) (28), with gender estimate call rate threshold set to 0.1, and BCFTools (29) used before conversion to variant call format (VCF) for further analyses in plink (30) and RStudio (31). After conversion to VCF, eight signal intensity parameters (SIPs) including Raw X (RawX), Raw Y (RawY), Normalized X (NormX), Normalized Y (NormY), Normalized R (NormR), Theta (*θ*), Log R Ratio (LRR), B-Allele Frequency (BAF), and the quality metric Illumina GenCall Confidence Score (IGC) were extracted for all SNPs using vcftools (32). SIPs extracted are described in Supplementary Table S1. Genotype call for each SNP was also extracted in accordance with the GSA proprietary SNP calling algorithm.

The workflow is presented in Figure 1. Initial quality control (QC) measures at the SNP-level excluded i) SNPs that failed to convert from raw idat format to VCF format; ii) multi-allelic SNPs, iii) SNPs with non-A/T/C/G nitrogen base, and iv) SNPs with ambiguous nitrogen base-pairing (A/T or C/G). After initial QC, each SNP was stratified to one of four groups (A/C, A/G, T/C and T/G) according to base-pairing. To ensure consistency in the designation of SNP orientation, the generalized nomenclature of “Allele A” and “Allele B” within the Illumina genotyping system, also known as the Illumina TOP/BOT strand naming convention, was adapted for the base-pairings considered (33). For base-pairings A/C and A/G, the “A” nitrogen base was assigned as “Allele A”, while the “C” and “G” nitrogen bases were assigned “Allele B”. For base-pairings T/C and T/G, the “T” nitrogen base was assigned as “Allele A”, while the “C” and “G” nitrogen bases were assigned “Allele B”.

**Figure 1:**
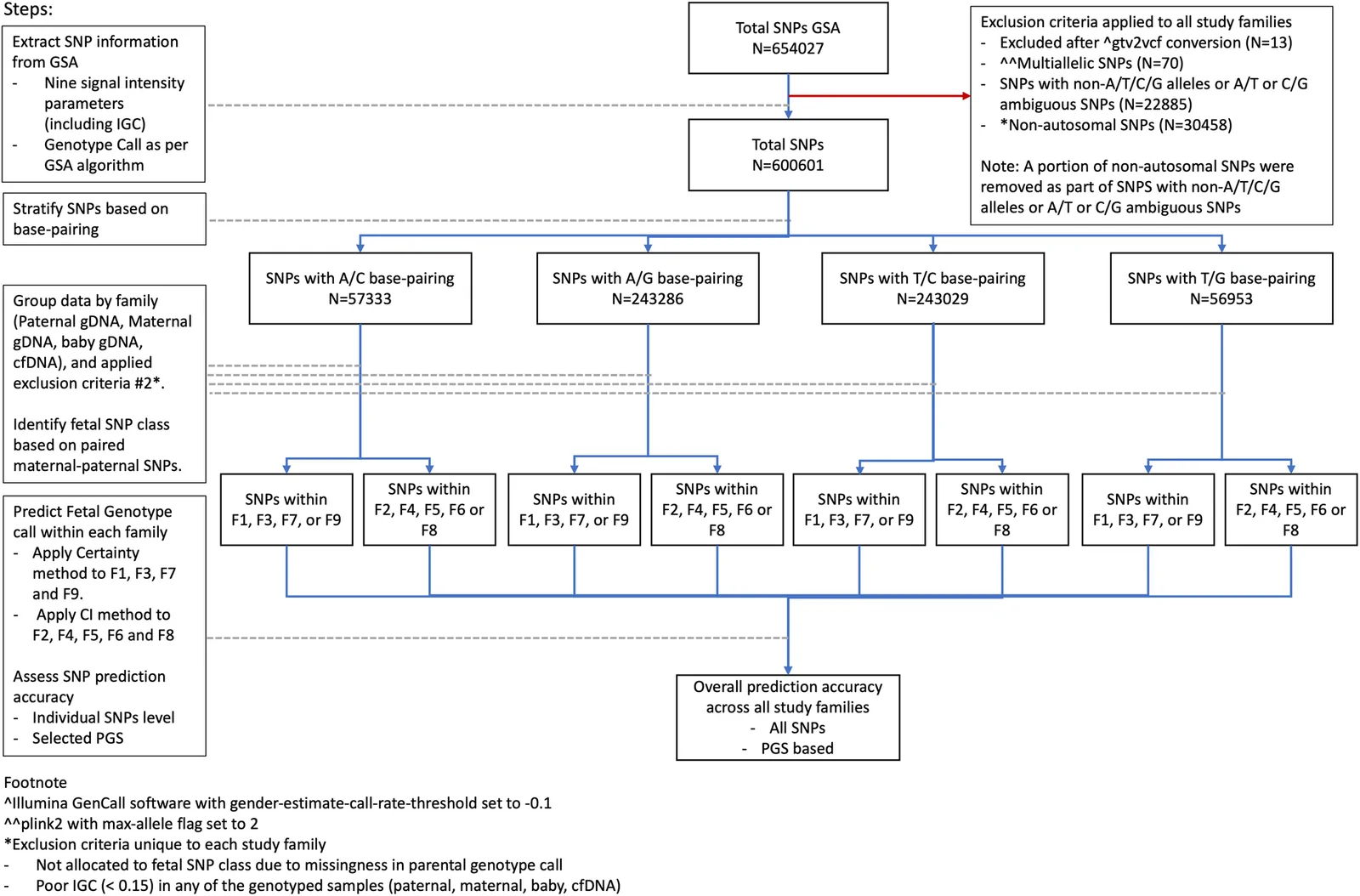
Methods flowchart.

Next, paired maternal-paternal SNPs, within each family unit, were examined to assign the corresponding SNP in T-cfDNA to one of nine possible fetal SNP classes F1 to F9 groups. The possible outcomes for fetal genotype based on paired maternal-paternal SNPs are illustrated in Figure 2. Fetal SNP classes F1, F3, F7 and F9 included SNPs where paired maternal-paternal SNPs are homozygous, implying only one possible outcome for fetal genotype. Fetal SNP classes F2, F4, F6 and F8 included paired maternal-paternal SNPs where one parent is homozygous while the other parent is heterozygous, implying two possible outcomes for fetal genotype. Lastly, fetal SNP class F5 included paired maternal-paternal SNPs where both parents are heterozygous, implying three possible outcomes for fetal genotype.

**Figure 2:**
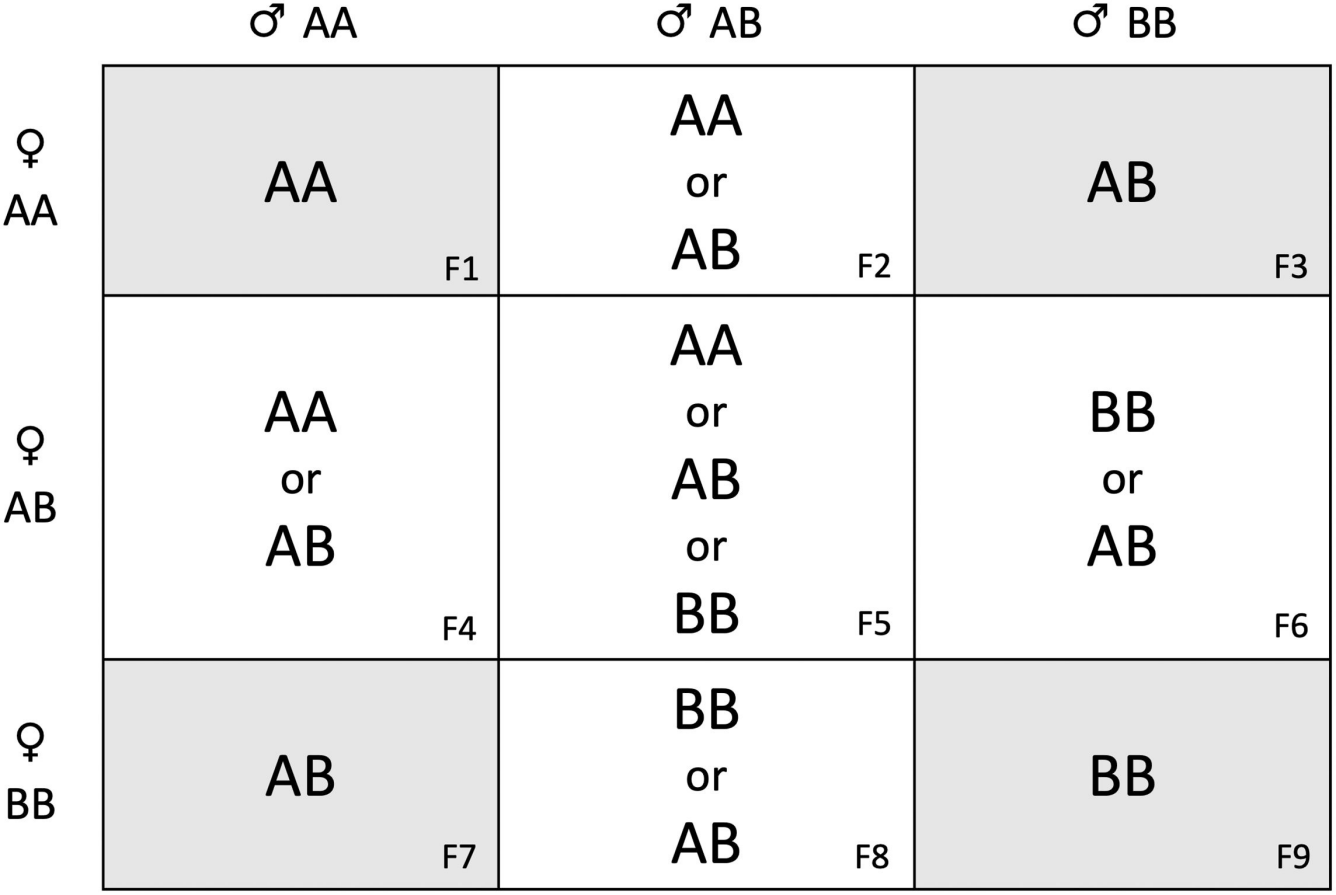
Maternal-Paternal combinations—possible fetal genotype call (s). Assignment of fetal SNP corresponding to paired maternal-paternal SNP. Paternal genotype calls are reflected in the columns (♂ AA, ♂ AB, and ♂ BB) while maternal genotype calls are reflected in the rows (♀ AA, ♀ AB, ♀ BB). Fetal SNPclasses are indicated in the lower right hand corner of each grid. The generalised nomenclature of “Allele A” and “Allele B” is used for illustrative purposes for all base-pairings considered (A/C, A/G, T/C and T/G).

Based on fetal SNP class, fetal genotypes were predicted either using the “Certainty” or the “Confidence Interval (CI)” method using SIPs extracted for T-cfDNA. The “Certainty” method was applied to SNPs in Fetal SNP classes F1, F3, F7 and F9 while the “CI” method was applied to the remaining SNPs.

The “CI” method involved comparing SIPs measured in cfDNA against the reference distributions for the three possible genotype calls (AA, AB and BB). Reference distributions could not be computed in instances where the genotype call in one parent was heterozygous. Hence, to maintain consistency in the source used to compute reference distributions for predicting fetal genotype calls from cfDNA, reference distributions were constructed using maternal SNPs. The “CI” method, illustrated in Figure 3, involved the comparison of each SNP SIP in T-cfDNA against the confidence interval of the SIPs constructed using maternal SNPs (based on maternal gDNA). Briefly, all SNPs in each individual study woman were examined and stratified to one of three groups: wildtype homozygous (matAA), heterozygous (matAB), and alternative allele homozygous (matBB). The 95% CIs for the three groups of SNPs for each study woman were then calculated via bootstrapping for all eight SIPs; the IGC parameter (<0.15) was used to identify SNPs for exclusion from further analyses. SNPs in T-cfDNA were stratified to one of the three groups based on its matching maternal SNP, and SIPs were compared against the 95% CI constructed for the corresponding group. The 95% CIs for each SIP for the three maternal genotype groups for maternal gDNA from participant from FPS-030 are presented in Supplementary Table S2 for illustration purposes. T-cfDNA SNPs in Fetal SNP classes F2 and F4 were examined against the 95% CI for SIPs constructed using matAA and matAB SNPs, respectively, to predict fetal genotype call “AA” vs. “AB”. T-cfDNA SNPs in Fetal SNP class F5 were examined against the 95% CIs for SIPs constructed using matAB SNPs to predict fetal genotype call “AA” vs. “AB” vs. “BB”. Lastly, T-cfDNA SNPs in Fetal SNP classes F6 and F8 were examined against the 95% CIs for SIPs constructed using matAB and matBB SNPs, respectively, to predict fetal genotype call “AB” vs. “BB”. Greater detail for the “CI” method is described in Figure 3. Predicted fetal SNP were examined against direct genotyping of cord blood for assessment of prediction accuracy. The 90% and 99% CIs for the three maternal SNP groups were also constructed to assess if predictive performance differed from the 95% CI.

**Figure 3:**
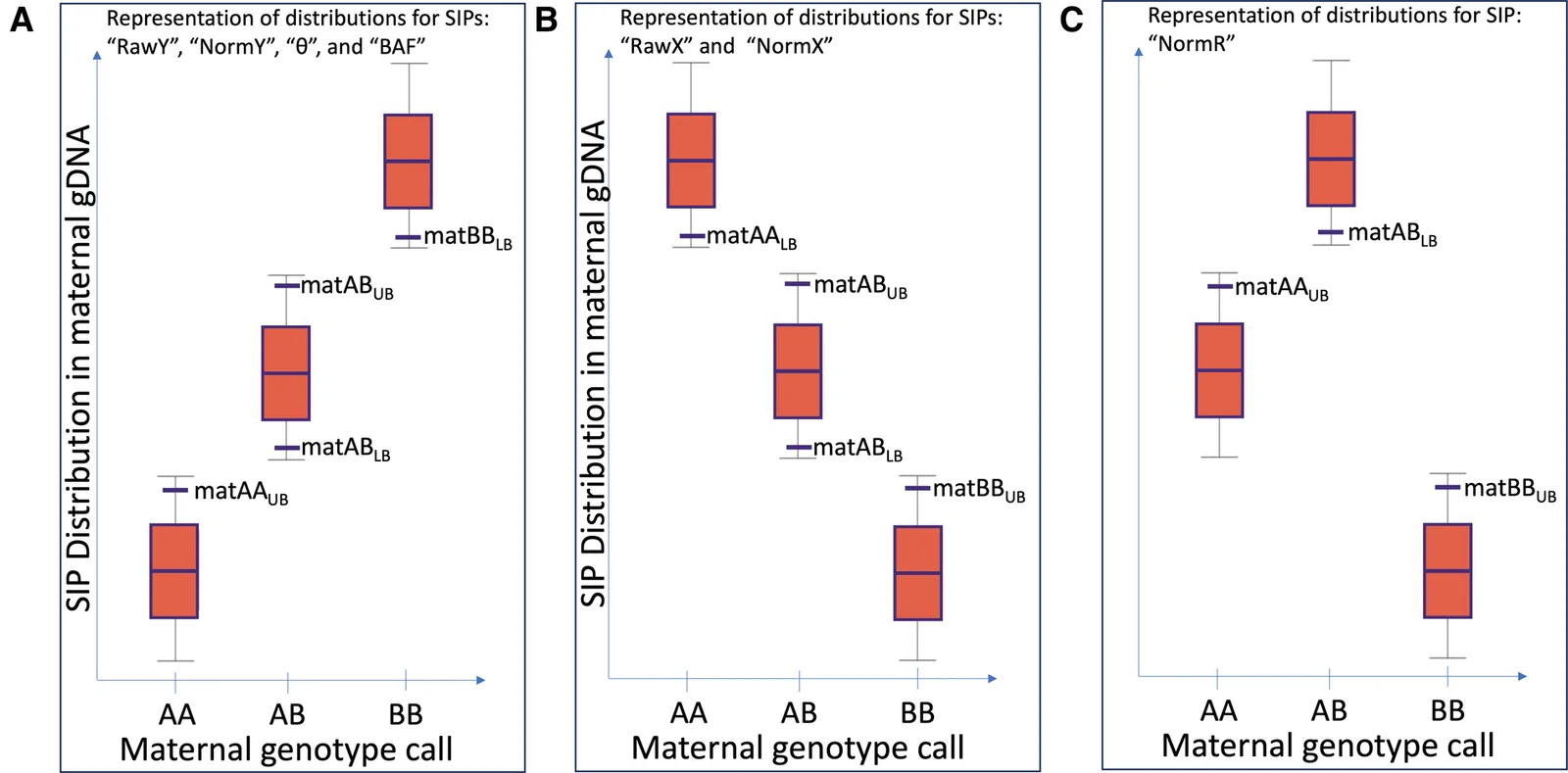
Illustration of the confidence interval method. Illustration of the “within Confidence Interval” method to predict fetal SNP genotype call; UB denotes upper bound; LB denotes lower bound. Not drawn to scale; these figures illustrate the trends observed for each SIP when comparing groups of SNPs within matAA, matAB and matBB. **(A)** Represents the distribution for maternal SIPs RawY, NormY, *θ*, and BAF: i. F2 SNPs, fetal genotype was assigned “AA” if the fetal SIP was less than the matAA_UB_, otherwise fetal genotype was assigned “AB”; ii. F4 SNPs, fetal genotype was assigned “AA” if the fetal SIP was less than the matAB_LB_, otherwise fetal genotype was assigned “AB”; iii. F5 SNPs, fetal genotype was assigned “AA” if the fetal SIP was less than the matAB_LB_, “AB” if the fetal SIP was between the matAB_LB_ and matAB_UB_, and “BB” if fetal SIP was greater than the matAB_UB_; iv. F6 SNPs, fetal genotype was assigned “BB” if the fetal SIP was greater than the matAB_UB_, otherwise fetal genotype was assigned “AB”; v. F8 SNPs, fetal genotype was assigned “BB” if the fetal SIP was greater than the matBB_LB_, otherwise fetal genotype was assigned “AB”. **(B)** Represents the distribution for maternal SIPs RawX and NormX, i. F2 SNPs, fetal genotype was assigned “AA” if the fetal SIP was less than the matAA_LB_, otherwise fetal genotype was assigned “AB”; ii. F4 SNPs, fetal genotype was assigned “AA” if the fetal SIP was greater than the matAB_UB_, otherwise fetal genotype was assigned “AB”; iii. F5 SNPs, fetal genotype was assigned “AA” if the fetal SIP was greater than the matAB_UB_, “AB” if the fetal SIP was between the matAB_LB_ and matAB_UB_, and “BB” if fetal SIP was less than the matAB_LB_; iv. F6 SNPs, fetal genotype was assigned “BB” if the fetal SIP was less than the matAB_LB_, otherwise fetal genotype was assigned “AB”; v. F8 SNPs, fetal genotype was assigned “BB” if the fetal SIP was less than the matBB_UB_, otherwise fetal genotype was assigned “AB”. **(C)** Represents the distribution for maternal SIP NormR, i. F2 SNPs, fetal genotype call was assigned “AA” if the fetal SIP was less than the matAA_UB_, otherwise fetal genotype call was assigned “AB”; ii. F4 SNPs, fetal genotype call was assigned “AA” if the fetal SIP was less than the matAB_LB_, otherwise fetal genotype call was assigned “AB”; iii. F5 SNPs, fetal genotype call was assigned “AA” if the fetal SIP was between the matBB_UB_ and matAB_LB_; fetal genotype call was assigned “AB” if the fetal SIP was greater than the matAB_LB_, otherwise, fetal genotype was assigned “BB” if the fetal SIP was less than the matBB_UB_. iv. F6 SNPs, fetal genotype call was assigned “BB” if the fetal SIP was less than the matAB_LB_, otherwise fetal genotype call was assigned “AB”; v. F8 SNPs, fetal genotype call was assigned “BB” if the fetal SIP was less than the matBB_UB_, otherwise fetal genotype call was assigned “AB”.

Next, the potential to apply the “Certainty” and “CI” methods to predict polygenic scores (PGSs) was assessed. Specifically, six PGSs, constructed with varying numbers of SNPs, for phenotypes spanning several different health domains were evaluated. These PGS including two birthweight PGSs (BW2016-PGS, 60 SNPs, and BW2019-PGS, 146 SNPs) (11, 17), depression PGS (DEP-PGS, 102 SNPS) (34), placental weight PGS (PW-PGS, 39 SNPs) (35), Attention Deficit/Hyperactivity Disorder PGS (ADHD-PGS, 27 SNPs) (36) and childhood obesity-PGS (ChOB-PGS, 22 SNPs) (37) were assessed. The SNPs used to derive these PGSs were identified through GWAS and candidate gene studies. Predicted PGSs were compared against PGS based on the SNPs directly genotyped from cord blood. Wilcoxon's paired test was used to assess for systematic biases in central tendency of the predicted PGS, while differences between predicted and actual PGSs were assessed using the coefficient of variation and presented graphically via Bland-Altman plots.

Finally, fetal fraction (FF) was estimated using T-cfDNA by expressing the signal of the alternative allele (with reference to the maternal homozygous genotype call) as a proportion of the sum of the A and B alleles signals in SNPs from Fetal SNP class F3, where maternal genotype call is homozygous and fetal genotype call is heterozygous (e.g., the signal contributed by the B allele in T-cfDNA as a proportion of the sum of the signal contributed by the A and B alleles in T-cfDNA). FF is hence estimated by computing the average expression of the SIP “NormY” as a percent of the sum of the SIPs “NormX” and “NormY” for the corresponding SNP. Sensitivity analyses were performed after excluding study families based on FF outliers by visual examination of the FF distribution.

All analyses were completed using iaap-cli (28), BCFTools (29), plink (30) and RStudio (31). All analytic code can be made available upon request.

## 3 Results

The mean T-cfDNA yield in the study population was 47.115 ng (standard deviation = 26.742 ng). Of the 30 families recruited, a total of 21 families met the T-cfDNA yield criteria of 25 ng or greater and remained in the final analytic dataset.

A total of 654,027 SNPs were directly genotyped on the Illumina Infinium Global Screening Array-24 v3.0 (GSA) Beadchip. After excluding SNPs that failed to convert from raw data to VCF format (*N* = 13), multiallelic SNPs (*N* = 70), SNPs with non-A/T/C/G alleles or ambiguous A/T or C/G base-pairing (*N* = 22,885), and non-autosomal SNPs (*N* = 30,458), a total of 600,601 SNPs remained for further analyses. Of these, there were a total of 57,333 A/C, 243,286 A/G, 243,029T/C, and 56,953T/G SNPs (shown in Figure 1).

Overall fetal SNP class distribution across the study families is presented in Figure 4; Fetal SNP class distribution by the individual SNP base-pairings is presented in Supplementary Figure S1. Most SNPs (72.40%) fall in SNP classes F1, F3, F7 and F9, and were predicted using the “Certainty” method. The remaining SNPs (27.60%) were predicted using the “CI” method.

**Figure 4:**
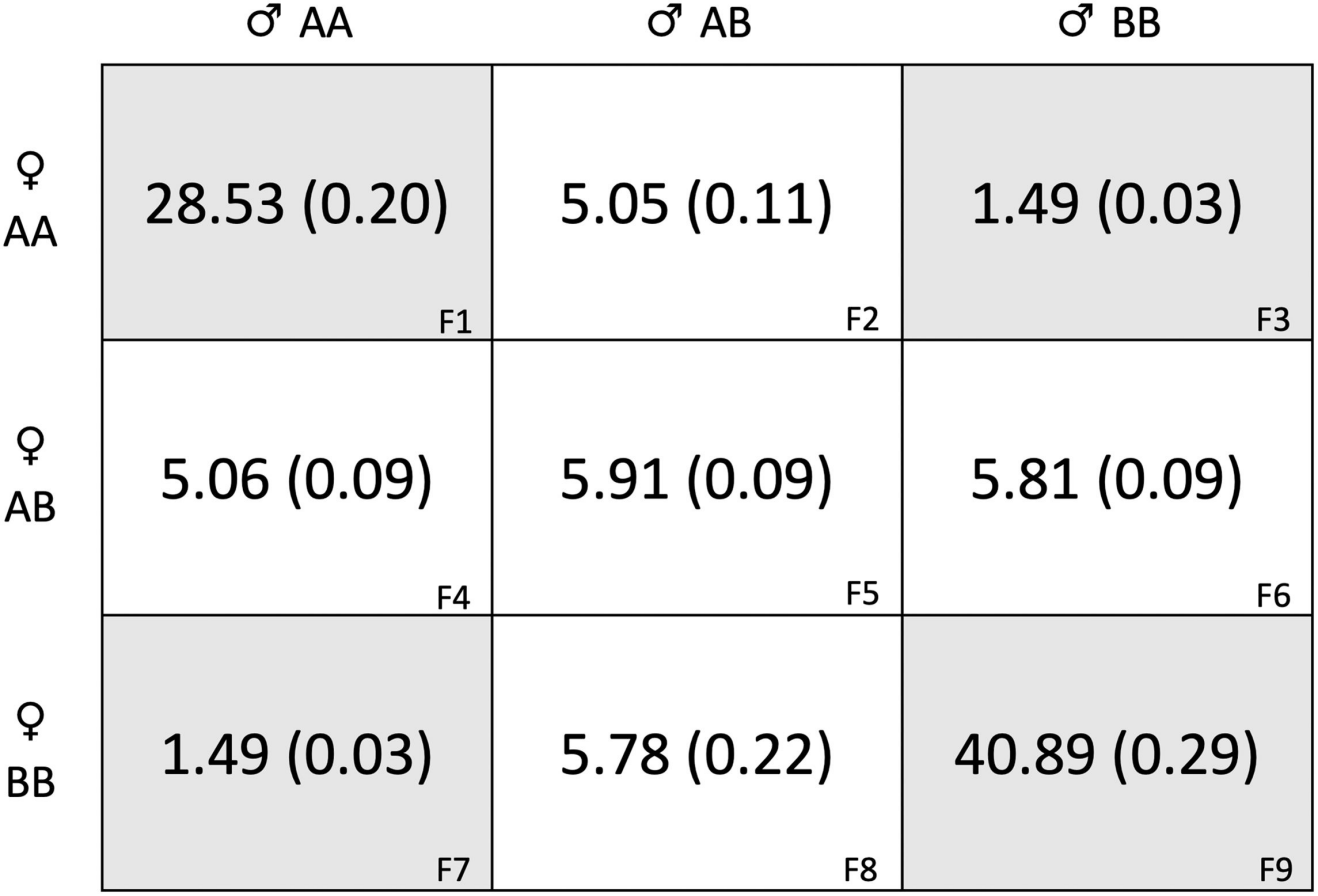
Mean and standard deviation of SNPclass distribution in the study population. Mean and standard deviation of the overall distribution of SNPs across the 21 study families.

Prediction accuracies for fetal SNPs in SNP classes F1, F3, F7 and F9 exceeded 99% across the study families. The mean percent accuracies were 99.998 [95% CI: 99.996–99.998], 99.881 [95% CI: 99.856–99.906], 99.852 [95% CI: 99.826–99.878], and 99.997 [95% CI: 99.994–99.999] for SNP classes F1, F3, F7 and F9, respectively. Prediction accuracies of the fetal SNPs across the remaining SNP classes for using the “CI” method were significantly lower and are presented in Table 1. Each SIP appeared to predict fetal SNPs in SNP class F2 with the highest accuracy, and fetal SNPs in SNP class F5 with the lowest accuracy. Amongst all SIPs examined, the SIP “BAF” predicted fetal SNPs in SNP classes F2, F4, and F6 with the highest accuracy. Next, the SIP “NormR” predicted fetal SNPs in SNP class F5 with the highest accuracy. Lastly, the SIP “*θ*” predicted fetal SNPs in SNP class F8 with the highest accuracy. The overall mean percent accuracy across all nine SNP classes was 86.39% (95% CI: 85.94%–86.84%) (Supplementary Table S3). Prediction accuracies for SNPs in Fetal SNP classes F2, F4, F5, F6 and F8 by individual SNP base-pairing (presented in Supplementary Tables S4–S7) were similar for all base-pairing patterns. The predictive performances for fetal SNPs in SNP classes using the 90% and 99% CIs are similar to those with the 95% CIs and are presented in Supplementary Tables S14–S19 and S26–S31, respectively.

**Table 1:** Mean and standard deviation for (percent) prediction accuracy across all study families for SNPs in SNPclasses F2, F4, F5, F6 and F8 (all SNP base-pairing) [95% CI].

SIP	F2	F4	F5	F6	F8	Overall accuracy
	Mean (SD)	Mean (SD)	Mean (SD)	Mean (SD)	Mean (SD)	Mean (SD)
RawX	49.98 (0.52) [49.74–50.22]	50.03 (0.44) [49.83–50.23]	25.30 (0.54) [25.05–25.55]	50.53 (0.49) [50.31–50.75]	53.49 (2.67) [52.27–54.71]	45.57 (0.76) [45.22–45.92]
RawY	55.29 (3.75) [53.58–57.00]	50.45 (0.37) [50.28–50.62]	25.53 (0.62) [25.25–25.81]	50.20 (0.40) [50.02–50.38]	49.99 (0.48) [49.77–50.21]	45.86 (0.89) [45.45–46.27]
NormX	50.11 (0.41) [49.92–50.30]	50.45 (0.44) [50.25–60.65]	25.55 (0.67) [25.25–25.85]	50.58 (0.48) [50.36–50.80]	56.70 (4.77) [54.53–58.87]	46.41 (1.31) [45.81–47.01]
NormY	58.21 (5.21) [55.84–60.58]	50.59 (0.36) [50.43–50.75]	25.66 (0.70) [25.34–25.98]	50.42 (0.51) [50.19–50.65]	50.14 (0.46) [49.94–50.35]	46.52 (1.27) [45.94–47.10]
NormR	50.11 (0.32) [49.96–50.26]	49.89 (0.27) [49.77–50.01]	35.85 (1.39) [35.22–36.48]	50.10 (0.36) [49.94–50.26]	50.14 (0.33) [49.99–50.29]	47.02 (0.34) [46.87–47.17]
*θ*	56.62 (5.11) [54.29–58.95]	52.41 (1.49) [51.73–53.09]	27.59 (1.72) [26.81–28.37]	52.39 (1.48) [51.72–53.06]	56.99 (5.54) [54.47–59.51]	48.84 (3.01) [47.47–50.21]
BAF	58.44 (4.98) [56.17–60.71]	53.55 (2.23) [52.53–54.57]	28.85 (2.60) [27.67–30.03]	53.86 (2.64) [52.66–55.06]	53.71 (2.36) [52.64–54.78]	49.27 (2.78) [48.00–50.54]
Best Predictor	BAF	BAF	normR	BAF	θ	

Next, predicted genotype calls for fetal SNPs were used to derive PGS's and assessed against PGS's based on cord blood. The SIP “BAF” was used to predict fetal SNPs in Fetal SNP classes F2, F4, and F6, the SIP “NormR” was used to predict fetal SNPs in Fetal SNP class F5; while the SIP “*θ*” was used to predict fetal SNPs in Fetal SNP class F8.

The PGS prediction accuracies for BW-PGS2019, Dep-PGS, BW2016-PGS, PW-PGS, ADHD-PGS, and ChOB-PGS, listed in decreasing order of number of SNPs in each PGS, are presented in Supplementary Tables S8–S13. No systematic bias in central tendency was demonstrated between predicted and actual PGSs in all six predicted PGS and their corresponding PGSs based on cord blood (all Wilcoxon's paired test *p*-values > 0.05). The Bland-Altman (BA) plots suggested poor agreement between predicted PGS and PGS based on cord blood (Figure 5). The coefficients of variation (%CV) for BW-PGS2019, Dep-PGS, BW2016-PGS, PW-PGS, ADHD-PGS, and ChOB-PGS were 5.61%, 7.00%, 7.88%, 6.33%, 12.90% and 16.47%, respectively and appeared to demonstrate an inverse relationship with the number of SNPs used for PGS computation (Supplementary Tables S8−S13). The predictive performances using the 90% and 99% CIs are similar and are presented in the Supplementary Section (90% CI Supplementary Tables S20–S25 and Figure S2; 99% CI Supplementary Tables S32–S37 and Figure S3).

**Figure 5:**
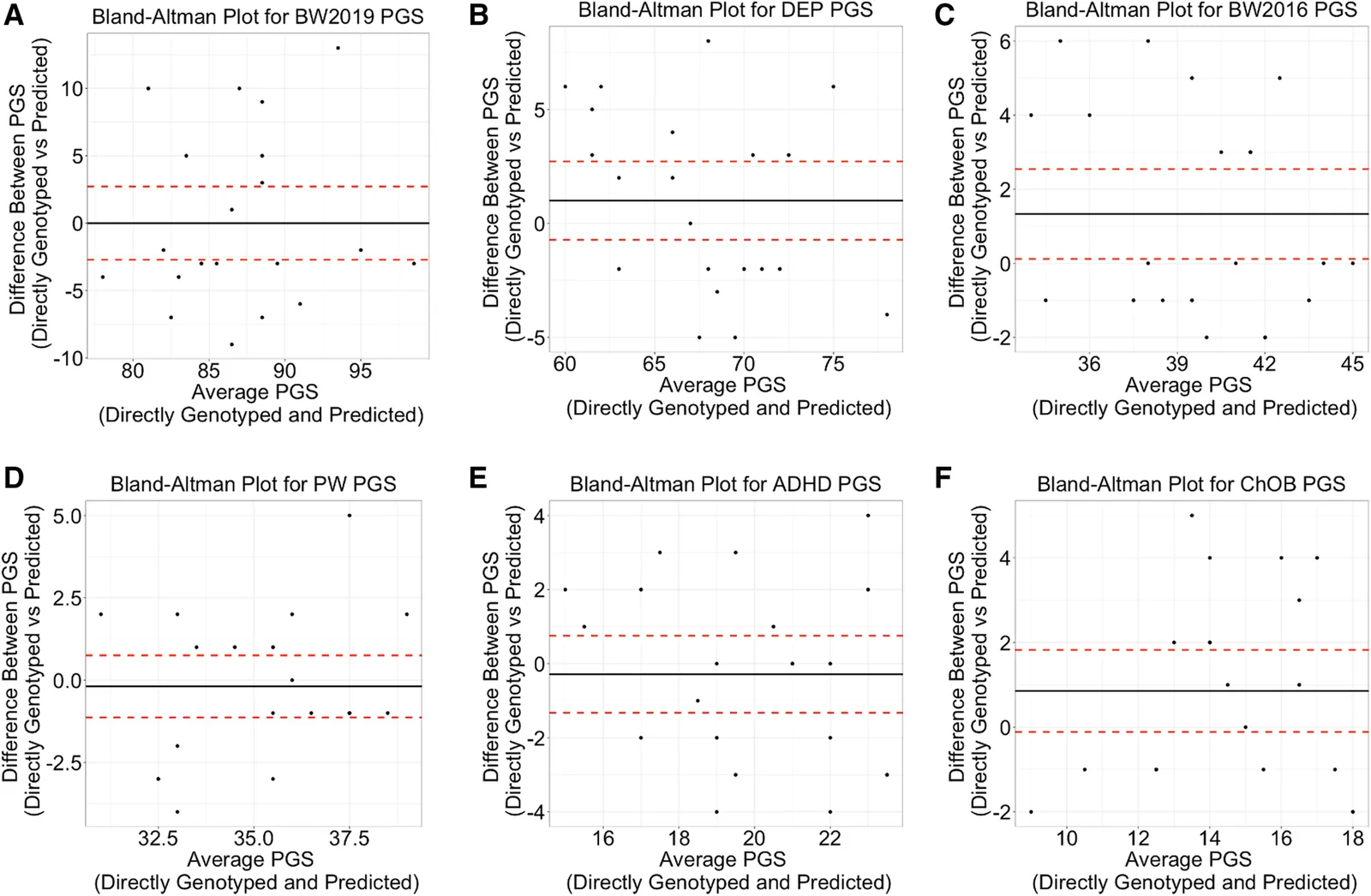
Graphical representation of discrepancies between PGS based on direct genotyping of cord blood vs. predicted from parental samples (certainty method + 95% CI for maternal SNPs) Bland-Altman plots to illustrate the discrepancies between polygenic scores (PGS)—Directly genotyped based on cord blood samples vs. Derived based on Certainty method + Confidence Interval (CI) method using the 95% CI. **(A)** Birthweight 2019 (BW2019-PGS); **(B)** depression (DEP-PGS); **(C)** birthweight 2016 (BW2016-PGS); **(D)** placental weight (PW-PGS); **(E)** attention deficit/hyperactivity disorder (ADHD-PGS); **(F)** childhood obesity (ChOB-PGS).

Five families were outliers based on their estimated fetal fraction (FF). The sensitivity analyses that followed the exclusion of these families did not demonstrate significant improvements in overall SNP prediction accuracy (86.80%, 95% CI: 86.44–87.14%). Further, except for the ADHD-PGS, there was an increase in CV across all other GSs examined in this study (%CV range: 5.92–17.62%). When prediction accuracies within individual Fetal SNP classes were examined, study families identified as outliers appeared to have lower prediction accuracy whereas increased accuracy was observed with increased FF in SNPs within Fetal SNP classes F2, F4, F6 and F8 (Supplementary Figures S4, S5).

## 4 Discussion

We developed a non-invasive method to predict fetal SNP genotype using paternal and maternal gDNA, and T-cfDNA from maternal circulation in pregnancy, and assessed its potential use in deriving polygenic scores for phenotypes that span several health domains. Based on SNPs with a minimum IGC ≥ 0.15, the mean overall prediction accuracy at the individual SNP level was 86.39% (95% CI: 85.94%–86.84%). Bland-Altman plots and coefficient of variation for the PGSs, however, suggested poor agreement and the need for a marked improvement in prediction accuracy before this approach could be utilized in a clinical setting. The mean overall prediction accuracy at the individual SNP level exceeded 99% where paired paternal and maternal SNPs were homozygous, which implies the predictive performance is limited when one of the parental SNPs is heterozygous.

The SIPs “*θ*” and “BAF” measure deviations from the homozygous call in a SNP; the SIPs “RawX” and “NormX” measure the presence of the TOP (or A) allele; the SIPs “RawY” and “NormY” measure the presence of the BOT (or B) allele; the “SIP” measures the presence of a balance between the A and B alleles. In SNP class F2 where maternal genotype calls are homozygous (AA), SIP “BAF” was the best predictor of fetal genotype, followed by the SIP “NormY” which corresponded to the SIP that measures the presence of the opposing B allele. Similarly, in SNP class F8 where maternal genotype calls are homozygous (BB), SIP “*θ*” was the best predictor of fetal genotype, followed by the SIP “NormX” which corresponded to the SIP that measures the presence of the opposing A allele. We also observed that prediction accuracy using the “CI” method was higher in SNP classes F2 and F8 compared to SNP classes F4, F5 and F6. These results suggest that the GSA is potentially marginally more sensitive in identifying discordance between maternal and fetal cfDNA in scenarios where there is an imbalance between the A and the B alleles (i.e., when maternal genotype call is homozygous) compared to when there is a balance between the A and B alleles (i.e., when maternal genotype call is heterozygous). The smaller magnitudes of deviation from the maternal SIP measures observed in T-cfDNA potentially contributed to the poorer predictive performance when maternal genotype call is heterozygous (Fetal SNP classes F4, F5 and F6). The enrichment and amplification of T-cfDNA such that the SNP discordance between maternal and fetal could reach the minimum threshold for detection may improve overall prediction accuracy when the confidence interval method is applied.

The interassay coefficients of variation (CV), when comparing predicted PGS against PGS based on cord blood, for all PGS predicted ranged between 5.61% and 16.47%. Despite the general recommendation that an interassay CV less than 10% is considered acceptable in a clinical environment, it is worth noting that small errors in PGSs could translate to a significant difference in its matched phenotype. Kani et al. reported an interassay CV of ≤2% when they compared their novel genotyping method against the existing gold standard for genotyping, suggesting significant improvements to the method developed in this study are required prior to its clinical implementation (38).

In current clinical laboratory practice, FF is crucial when interpreting NIPT results for potential fetal chromosomal anomalies (22). In the clinical environment, NIPT results are not released when FF is low and repeat testing is usually recommended. In this study, we identified study families that had low FF level: these families were considered outliers. The exclusion of these families resulted in minimal improvement in overall mean percent accuracy across the study families when predicting fetal genotype using the “CI” method for SNPs in Fetal SNP classes F2, F4, F6 and F8. It is worth noting that the method used to estimate FF in this study is an approximation. Similar to other approximation algorithms used in clinical laboratory settings, variation exists when compared to gold standard approaches based on the Y-chromosome which is limited to male fetuses (22).

The strengths of this study include the stringent laboratory and bioinformatics protocols used to ensure data integrity, reliability, and reproducibility of the results. Our study was, however, limited by the small sample size and we recognize our results may lack precision and may not be generalizable to a different study population. A further limitation is that the T-cfDNA samples were collected at 36 weeks' gestation. We acknowledge that maternal sample collection for cfDNA collection occurred late in pregnancy as a substudy of the much larger NEW1000 pregnancy cohort study; this was to maximize the chances of success for this “proof of concept” study. Ideally, one would like this method to provide results early in pregnancy so that estimated personalized birthweight can be ascertained sufficiently early and deviations from this anticipated trajectory can potentially be investigated and treated quickly. Our results were promising and demonstrated potential feasibility to derive fetal genotype from parental samples. Future work should examine whether this method could be applied earlier in pregnancy, preferably around 10 weeks' gestation.

The literature suggests that the proportion of cell-free fetal DNA increases as pregnancy progresses (22, 39, 40). It is therefore possible that the performance of the “CI” method may be worse when applied to T-cfDNA samples collected earlier in pregnancy.

This study has provided promising initial results for a safe, non-invasive, economical approach to determine multiple fetal SNPs simultaneously. Despite an overall accuracy greater than 86% at the individual SNP level, the algorithm performed poorly when used to predict polygenic scores where the compounded effect of SNPs with allele frequencies between 20% and 40% were examined. While the accuracy attained in this study is modest, we have successfully demonstrated the potential feasibility of deriving fetal individual SNP genotype and developing PGSs from parental gDNA and maternal cfDNA in pregnancy. It is crucial to note that this study does not seek to develop a method to replace, nor is it equivalent to, existing non-invasive screening tests in pregnancy for aneuploidy, which target over 10,000 SNPs across several regions to evaluate the risks of common genetic (commonly at the chromosomal level) anomalies.

Due to the vast quantity of data obtained in SNP arrays, there is potential in applying more advanced methods to predict fetal genotype calls from parental samples during pregnancy. The proprietary method utilised by Illumina for assigning genotype calls on a SNP array is based on normalizing raw SIPs measures, and mapping the normalized coordinates against pre-determined references for the possible genotype calls (AA, AB and BB). Future work could explore if a multivariate/probabilistic classification method using multiple SIPs could enhance prediction outcomes. Other approaches to improve the accuracy of fetal SNP prediction include amplification of the fetal component in T-cfDNA or more advanced bioinformatic approaches including imputation and haplotype analyses.

## Data Availability

The datasets presented in this article are not readily available because of legal and ethical constraints. Requests to access the datasets should be made directly to the NEW1000 Study Management Group and Executive Committee (new1000@newcastle.edu.au).
