# Supplementary material for: A bespoke non-invasive approach to predict fetal genotype in pregnancy
Source: Front Med (Lausanne). 2026 Sep 28;13:1745404. doi: 10.3389/fmed.2026.1745404 (PMC13655113; doi:10.3389/fmed.2026.1745404)
Supplement: Supplementary file 1 [file Table1.docx]

***Supplementary Material***

**Supplementary Table 1.** Description of Signal Intensity Parameters (SIPs) from the Illumina Global Screening Array

| SIP | Description |
| --- | --- |
| RawX | Raw intensity of the A allele |
| RawY | Raw intensity of the B allele |
| NormX | Normalised intensity of the A allele |
| NormY | Normalised intensity of the B allele |
| NormR | Normalised intensity given by normR = normX + normY |
| Theta (θ) | Allelic intensity ratio given by θ = 2/ϖ*arctan($\frac{normY}{normX}$) |
| B-Allele Frequency (BAF) | Calibrated θ value that represents the proportion of B allele |
| Illumina GenCall confidence score (IGC) | SNP quality score |
| Log R Ratio (LRR) | Log R ratio |

**Supplementary Table 2.** 95% CI for SNP Signal Intensity Parameters (SIPs) for maternal homozygous (AA), maternal heterozygous (AB), and maternal alternative homozygous (BB) SNPs from mother from FPS-030

| Signal Intensity Parameter | Maternal Genotype Call | A/C SNPs  N_SNPs_=56415 | A/G SNPs  N_SNPs_=241787 | T/C SNPs  N_SNPs_=242083 | T/G SNPs  N_SNPs_=55823 |
| --- | --- | --- | --- | --- | --- |
| RawX | AA | 14621.23 (14550.62,14691.84) | 14968.46  (14932.43, 15004.49) | 14905.43  (14869.48, 14941.39) | 14578.76  (14506.59, 14650.94) |
|  | AB | 9570.21  (9495.19,9645.23) | 9832.71  (9795.93, 9869.51) | 9784.79  (9747.91, 9821.68) | 9503.98  (9430.38, 9577.59) |
|  | BB | 1124.27  (1117.66,1130.88) | 1169.62  (1166.30, 1172.94) | 1165.66  (1162.36, 1168.95) | 1125.10  (1118.71, 1131.49) |
| RawY | AA | 505.80  (501.50, 510.11) | 503.61  (501.45, 505.78) | 499.59  (497.45, 501.72) | 496.08  (491.90, 500.26) |
|  | AB | 4452.41  (4423.53, 4481.29) | 4591.01  (4576.96, 4605.05) | 4559.89  (4545.76, 4574.02) | 4477.34  (4448.72, 4505.97) |
|  | BB | 6989.95  (6962.29, 7017.61) | 7209.80  (7197.01, 7222.59) | 7180.68  (7167.87, 7193.49) | 7025.71  (6998.13, 7053.29) |
| NormX | AA | 0.999  (0.994, 1.004) | 1.0228  (1.0204, 1.0253) | 1.0186  (1.0162, 1.0211) | 0.9957  (0.9908, 1.0006) |
|  | AB | 0.625  (0.620, 0.630) | 0.6427  (0.6403, 0.6451) | 0.63922  (0.6368, 0.6417) | 0.6205  (0.6156, 0.6253) |
|  | BB | 0.04969  (0.04925, 0.05013) | 0.05220  (0.05198, 0.05243) | 0.05201  (0.05179, 0.05223) | 0.04976  (0.04934, 0.0502) |
| NormY | AA | 0.05143  (0.05086, 0.05199) | 0.05096  (0.05068, 0.05124) | 0.05054  (0.05026, 0.05082) | 0.05014  (0.04960, 0.05069) |
|  | AB | 0.59496  (0.59115, 0.59877) | 0.61413  (0.61227, 0.61600) | 0.6097  (0.6079, 0.6116) | 0.5986  (0.5948, 0.6024) |
|  | BB | 0.95501  (0.95143, 0.95860) | 0.9887  (0.9870, 0.9904) | 0.9847  (0.9830, 0.9864) | 0.9613  (0.9577, 0.9649) |
| NormR | AA | 1.0501  (1.0452, 1.0551) | 1.0738  (1.0713, 1.0763) | 1.0692  (1.0666, 1.0717) | 1.0458  (1.0408, 1.0509) |
|  | AB | 1.2197  (1.2116, 1.2279) | 1.2569  (1.2527, 1.2610) | 1.2490  (1.2449, 1.2531) | 1.2191  (1.2111, 1.2271) |
|  | BB | 1.0047  (1.0010, 1.0084) | 1.0409  (1.0392, 1.0427) | 1.0367  (1.0350, 1.0385) | 1.01110  (1.0073, 1.0148) |
| θ | AA | 0.03571  (0.03533, 0.03608) | 0.0344  (0.0342, 0.0345) | 0.03435  (0.03417, 0.03453) | 0.03511  (0.03474, 0.03550) |
|  | AB | 0.49330  (0.49153, 0.49507) | 0.4936  (0.4930, 0.4943) | 0.49320  (0.49256, 0.49384) | 0.49724  (0.49547, 0.49901) |
|  | BB | 0.96411  (0.96378, 0.96444) | 0.9642  (0.9641, 0.9644) | 0.96412  (0.96398, 0.96427) | 0.96447  (0.96416, 0.96478) |
| BAF | AA | -0.00597  (-0.00633, -0.005611) | -0.00547  (-0.005640, -0.005294) | -0.005776  (-0.005954, -0.005598) | -0.00624  (-0.006611, -0.005878) |
|  | AB | 0.51444  (0.51377, 0.51511) | 0.5156  (0.5153, 0.5160) | 0.5156  (0.5153, 0.5159) | 0.51492  (0.51424, 0.51560) |
|  | BB | 1.02126  (1.02081, 1.02172) | 1.0196  (1.0194, 1.0198) | 1.0198  (1.0196, 1.0200) | 1.0210  (1.0205, 1.0215) |

**Supplementary Table 3.** Overall mean and standard deviation for (percent) prediction accuracy of all SNPs (Fetal SNPclasses F1 to F9) predicted with the use of certainty method [F1, F3, F7 and F9] and 95% CI for maternal SNPs SIP [“BAF” for SNPs in Fetal SNPclasses F2, F4, and F6; SIP “NormR” for SNPs in Fetal SNPclass F5; SIP “θ” for SNPs in Fetal SNPclass F8]

| FamID | A/C SNPs | A/G SNPs | C/T SNPs | T/G SNPs | All  Base-Pairings |
| --- | --- | --- | --- | --- | --- |
| FPS001M | 86.14 | 86.56 | 86.39 | 86.10 | 86.41 |
| FPS002M | 86.52 | 86.80 | 86.83 | 86.32 | 86.74 |
| FPS003M | 86.83 | 87.16 | 87.03 | 86.67 | 87.03 |
| FPS005M | 85.30 | 85.73 | 85.66 | 85.20 | 85.61 |
| FPS006M | 86.84 | 87.30 | 87.16 | 86.79 | 87.15 |
| FPS008M | 85.93 | 86.14 | 86.01 | 86.02 | 86.06 |
| FPS009M | 85.10 | 85.40 | 85.29 | 85.16 | 85.30 |
| FPS011M | 87.18 | 87.67 | 87.46 | 87.10 | 87.48 |
| FPS012M | 86.88 | 87.54 | 87.43 | 87.14 | 87.40 |
| FPS013M | 85.80 | 86.32 | 86.38 | 85.83 | 86.25 |
| FPS014M | 86.91 | 87.28 | 87.19 | 86.76 | 87.16 |
| FPS015M | 87.53 | 88.14 | 87.89 | 87.62 | 87.93 |
| FPS016M | 86.00 | 86.70 | 86.54 | 86.32 | 86.53 |
| FPS019M | 87.21 | 87.84 | 87.62 | 87.21 | 87.63 |
| FPS022M | 84.97 | 85.62 | 85.44 | 85.05 | 85.43 |
| FPS025M | 85.04 | 85.50 | 85.23 | 84.83 | 85.28 |
| FPS026M | 84.46 | 85.04 | 84.70 | 84.41 | 84.79 |
| FPS027M | 85.19 | 85.56 | 85.59 | 85.17 | 85.50 |
| FPS028M | 84.51 | 84.69 | 84.59 | 84.36 | 84.60 |
| FPS029M | 86.70 | 86.91 | 86.74 | 86.44 | 86.78 |
| FPS030M | 86.60 | 87.32 | 87.15 | 86.87 | 87.14 |

**Supplementary Table 4.** Mean and standard deviation for (percent) prediction accuracy across all study families for A/C SNPs in SNPclasses F2, F4, F5, F6 and F8 predicted with 95% CI for maternal SNPs

| SIP | F2 | F4 | F5 | F6 | F8 | Overall Accuracy |
| --- | --- | --- | --- | --- | --- | --- |
|  | Mean (SD) | Mean (SD) | Mean (SD) | Mean (SD) | Mean (SD) | Mean (SD) |
| RawX | 50.16 (0.82) | 50.00 (0.87) | 25.47 (0.83) | 50.90 (1.01) | 53.15 (2.49) | 45.56 (0.82) |
| RawY | 55.34 (3.55) | 50.22 (0.74) | 25.79 (0.99) | 50.35 (0.76) | 50.21 (0.86) | 45.93 (0.77) |
| NormX | 50.16 (0.84) | 50.18 (1.12) | 25.80 (1.03) | 50.91 (0.91) | 56.55 (4.85) | 46.36 (1.47) |
| NormY | 58.06 (5.06) | 50.29 (1.05) | 25.98 (1.07) | 50.55 (0.87) | 50.12 (1.05) | 46.51 (1.21) |
| NormR | 50.21 (0.78) | 49.99 (1.00) | 35.65 (1.53) | 50.21 (0.96) | 50.09 (0.86) | 47.00 (0.56) |
| θ | 56.17 (5.00) | 51.89 (1.32) | 27.59 (1.63) | 52.25 (1.12) | 56.95 (5.66) | 48.55 (2.77) |
| BAF | 58.17 (4.92) | 53.54 (2.24) | 29.18 (2.56) | 53.91 (2.56) | 53.52 (2.21) | 49.22 (2.73) |
| Best Predictor | BAF | BAF | NormR | BAF | θ |  |

**Supplementary Table 5.** Mean and standard deviation for (percent) prediction accuracy across all study families for A/G SNPs in SNPclasses F2, F4, F5, F6 and F8 predicted with 95% CI for maternal SNPs

| SIP | F2 | F4 | F5 | F6 | F8 | Overall Accuracy |
| --- | --- | --- | --- | --- | --- | --- |
|  | Mean (SD) | Mean (SD) | Mean (SD) | Mean (SD) | Mean (SD) | Mean (SD) |
| RawX | 50.11 (0.75) | 49.89 (0.55) | 25.22 (0.52) | 50.50 (0.55) | 53.52 (2.71) | 45.58 (0.74) |
| RawY | 55.44 (3.80) | 50.63 (0.46) | 25.45 (0.74) | 50.23 (0.51) | 49.81 (0.63) | 45.89 (0.96) |
| NormX | 50.11 (0.59) | 50.47 (0.64) | 25.54 (0.69) | 50.48 (0.56) | 56.91 (4.88) | 46.47 (1.32) |
| NormY | 58.22 (5.18) | 50.67 (0.51) | 25.55 (0.78) | 50.42 (0.69) | 50.10 (0.58) | 46.53 (1.27) |
| NormR | 50.10 (0.59) | 49.88 (0.55) | 35.98 (1.39) | 50.08 (0.53) | 50.22 (0.41) | 47.07 (0.33) |
| θ | 56.71 (5.14) | 52.70 (1.62) | 27.66 (1.81) | 52.42 (1.60) | 57.06 (5.61) | 48.97 (3.10) |
| BAF | 58.77 (5.12) | 53.69 (2.33) | 28.82 (2.65) | 53.88 (2.72) | 53.84 (2.44) | 49.40 (2.85) |
| Best Predictor | BAF | BAF | NormR | BAF | θ |  |

**Supplementary Table 6.** Mean and standard deviation for (percent) prediction accuracy across all study families for T/C SNPs in SNPclasses F2, F4, F5, F6 and F8 predicted with 95% CI for maternal SNPs

| SIP | F2 | F4 | F5 | F6 | F8 | Overall Accuracy |
| --- | --- | --- | --- | --- | --- | --- |
|  | Mean (SD) | Mean (SD) | Mean (SD) | Mean (SD) | Mean (SD) | Mean (SD) |
| RawX | 49.86 (0.51) | 50.10 (0.59) | 25.36 (0.67) | 50.50 (0.61) | 53.54 (2.73) | 45.58 (0.81) |
| RawY | 55.21 (3.80) | 50.47 (0.54) | 25.48 (0.56) | 50.19 (0.45) | 50.05 (0.50) | 45.84 (0.86) |
| NormX | 50.09 (0.49) | 50.42 (0.41) | 25.53 (0.72) | 50.52 (0.56) | 56.56 (4.72) | 46.36 (1.30) |
| NormY | 58.21 (5.25) | 50.68 (0.51) | 25.66 (0.59) | 50.43 (0.55) | 50.18 (0.53) | 46.54 (1.28) |
| NormR | 50.11 (0.33) | 50.02 (0.35) | 35.79 (1.40) | 50.04 (0.44) | 50.09 (0.34) | 47.01 (0.35) |
| θ | 56.66 (5.18) | 52.38 (1.50) | 27.56 (1.72) | 52.49 (1.60) | 57.00 (5.53) | 48.85 (3.03) |
| BAF | 58.15 (4.83) | 53.46 (2.17) | 28.81 (2.57) | 53.90 (2.64) | 53.66 (2.37) | 49.18 (2.73) |
| Best Predictor | NormY | BAF | NormR | BAF | θ |  |

**Supplementary Table 7.** Mean and standard deviation for (percent) prediction accuracy across all study families for T/G SNPs in SNPclasses F2, F4, F5, F6 and F8 predicted with 95% CI for maternal SNPs

| SIP | F2 | F4 | F5 | F6 | F8 | Overall Accuracy |
| --- | --- | --- | --- | --- | --- | --- |
|  | Mean (SD) | Mean (SD) | Mean (SD) | Mean (SD) | Mean (SD) | Mean (SD) |
| RawX | 49.73 (0.89) | 50.37 (0.90) | 25.25 (0.81) | 50.36 (1.09) | 53.53 (2.76) | 45.48 (0.75) |
| RawY | 54.99 (3.75) | 49.87 (0.76) | 25.85 (1.01) | 49.95 (0.82) | 50.27 (1.02) | 45.74 (0.99) |
| NormX | 50.12 (0.90) | 50.81 (0.75) | 25.43 (1.13) | 50.95 (0.99) | 56.55 (4.64) | 46.41 (1.27) |
| NormY | 58.29 (5.51) | 50.22 (0.58) | 25.83 (1.12) | 50.18 (1.03) | 50.15 (0.70) | 46.45 (1.40) |
| NormR | 50.05 (0.92) | 49.33 (0.66) | 35.74 (1.71) | 50.38 (0.83) | 50.03 (0.88) | 46.89 (0.52) |
| θ | 56.55 (5.11) | 51.90 (1.64) | 27.44 (1.86) | 51.99 (1.60) | 56.67 (5.36) | 48.49 (2.85) |
| BAF | 58.55 (5.34) | 53.36 (2.41) | 28.81 (2.78) | 53.51 (2.69) | 53.55 (2.55) | 49.12 (2.83) |
| Best Predictor | BAF | BAF | NormR | BAF | θ |  |

**Supplementary Tables 8-13** present the comparison of predicted PGS (based on genotype call derived from the certainty and maternal 95% CI SNPs) and PGS based on cord blood. The number of SNPs identified in the published literature for each phenotype is listed before the start of each table.

**Supplementary Table 8.** BW-PGS2019 [NumSNPs=146]

| FamID | Num SNPs available  [PGS range*] | PGS based on cord blood genotype | PGS based on prediction | Difference between cord blood and predicted PGS | Mean PGS –  Cord blood and predicted |
| --- | --- | --- | --- | --- | --- |
| FPS001M | 74 [0 - 148] | 88 | 91 | -3 | 89.5 |
| FPS002M | 75 [0 - 150] | 86 | 81 | 5 | 83.5 |
| FPS003M | 76 [0 - 152] | 76 | 80 | -4 | 78 |
| FPS005M | 76 [0 - 152] | 91 | 86 | 5 | 88.5 |
| FPS006M | 76 [0 - 152] | 83 | 86 | -3 | 84.5 |
| FPS008M | 74 [0 - 148] | 81 | 83 | -2 | 82 |
| FPS009M | 75 [0 - 150] | 79 | 86 | -7 | 82.5 |
| FPS011M | 76 [0 - 152] | 90 | 87 | 3 | 88.5 |
| FPS012M | 75 [0 - 150] | 92 | 82 | 10 | 87 |
| FPS013M | 75 [0 - 150] | 86 | 76 | 10 | 81 |
| FPS014M | 76 [0 - 152] | 97 | 100 | -3 | 98.5 |
| FPS015M | 76 [0 - 152] | 81 | 85 | -4 | 83 |
| FPS016M | 75 [0 - 150] | 84 | 87 | -3 | 85.5 |
| FPS019M | 76 [0 - 152] | 83 | 86 | -3 | 84.5 |
| FPS022M | 76 [0 - 152] | 94 | 96 | -2 | 95 |
| FPS025M | 75 [0 - 150] | 88 | 94 | -6 | 91 |
| FPS026M | 75 [0 - 150] | 100 | 87 | 13 | 93.5 |
| FPS027M | 72 [0 - 144] | 87 | 86 | 1 | 86.5 |
| FPS028M | 73 [0 - 146] | 82 | 91 | -9 | 86.5 |
| FPS029M | 75 [0 - 150] | 85 | 92 | -7 | 88.5 |
| FPS030M | 75 [0 - 150] | 93 | 84 | 9 | 88.5 |
| Mean (SD) | | | | | 86.952 (4.876) |
| % Coefficient of Variation (CV) | | | | | 5.61 |
| P-value for Wilcoxon’s Paired Test | | | | | 0.862 |

*PGS range based on the number of genotyped SNPs available on the GSA for each family for PGS computation

**Supplementary Table 9**. Dep-PGS [NumSNPs=102]

| FamID | Num SNPs available  [PGS range*] | PGS based on cord blood genotype | PGS based on prediction | Difference between cord blood and predicted PGS | Mean PGS –  Cord blood and predicted |
| --- | --- | --- | --- | --- | --- |
| FPS001M | 63 [0 - 126] | 72 | 69 | 3 | 70.5 |
| FPS002M | 60 [0 - 120] | 68 | 64 | 4 | 66 |
| FPS003M | 63 [0 - 126] | 74 | 71 | 3 | 72.5 |
| FPS005M | 63 [0 - 126] | 62 | 64 | -2 | 63 |
| FPS006M | 63 [0 - 126] | 78 | 72 | 6 | 75 |
| FPS008M | 59 [0 - 118] | 63 | 57 | 6 | 60 |
| FPS009M | 62 [0 - 124] | 69 | 71 | -2 | 70 |
| FPS011M | 62 [0 - 124] | 67 | 70 | -3 | 68.5 |
| FPS012M | 63 [0 - 126] | 67 | 67 | 0 | 67 |
| FPS013M | 62 [0 - 124] | 63 | 60 | 3 | 61.5 |
| FPS014M | 62 [0 - 124] | 65 | 59 | 6 | 62 |
| FPS015M | 63 [0 - 126] | 76 | 80 | -4 | 78 |
| FPS016M | 62 [0 - 124] | 64 | 62 | 2 | 63 |
| FPS019M | 63 [0 - 126] | 67 | 72 | -5 | 69.5 |
| FPS022M | 63 [0 - 126] | 67 | 65 | 2 | 66 |
| FPS025M | 63 [0 - 126] | 67 | 69 | -2 | 68 |
| FPS026M | 62 [0 - 124] | 65 | 70 | -5 | 67.5 |
| FPS027M | 63 [0 - 126] | 70 | 72 | -2 | 71 |
| FPS028M | 60 [0 - 120] | 72 | 64 | 8 | 68 |
| FPS029M | 62 [0 - 124] | 64 | 59 | 5 | 61.5 |
| FPS030M | 62 [0 - 124] | 71 | 73 | -2 | 72 |
| Mean (SD) | | | | | 67.643 (4.733) |
| % Coefficient of Variation (CV) | | | | | 7.00 |
| P-value for Wilcoxon Paired Test | | | | | 0.222 |

* PGS range based on the number of genotyped SNPs available on the GSA for each family for PGS computation

**Supplementary Table 10**. BW-PGS2016 [NumSNPs=60]

| FamID | Num SNPs available  [PGS range*] | PGS based on cord blood genotype | PGS based on prediction | Difference between cord blood and predicted PGS | Mean PGS –  Cord blood and predicted |
| --- | --- | --- | --- | --- | --- |
| FPS001M | 31 [0 - 62] | 43 | 40 | 3 | 41.5 |
| FPS002M | 31 [0 - 62] | 38 | 34 | 4 | 36 |
| FPS003M | 32 [0 - 64] | 38 | 39 | -1 | 38.5 |
| FPS005M | 32 [0 - 64] | 41 | 43 | -2 | 42 |
| FPS006M | 32 [0 - 64] | 38 | 32 | 6 | 35 |
| FPS008M | 32 [0 - 64] | 39 | 41 | -2 | 40 |
| FPS009M | 32 [0 - 64] | 34 | 35 | -1 | 34.5 |
| FPS011M | 32 [0 - 64] | 41 | 35 | 6 | 38 |
| FPS012M | 31 [0 - 62] | 37 | 38 | -1 | 37.5 |
| FPS013M | 31 [0 - 62] | 38 | 38 | 0 | 38 |
| FPS014M | 32 [0 - 64] | 41 | 43 | -2 | 42 |
| FPS015M | 32 [0 - 64] | 42 | 39 | 3 | 40.5 |
| FPS016M | 31 [0 - 62] | 36 | 32 | 4 | 34 |
| FPS019M | 32 [0 - 64] | 42 | 37 | 5 | 39.5 |
| FPS022M | 32 [0 - 64] | 44 | 44 | 0 | 44 |
| FPS025M | 32 [0 - 64] | 39 | 40 | -1 | 39.5 |
| FPS026M | 31 [0 - 62] | 41 | 41 | 0 | 41 |
| FPS027M | 32 [0 - 64] | 45 | 45 | 0 | 45 |
| FPS028M | 32 [0 - 64] | 43 | 40 | 3 | 41.5 |
| FPS029M | 32 [0 - 64] | 43 | 44 | -1 | 43.5 |
| FPS030M | 32 [0 - 64] | 45 | 40 | 5 | 42.5 |
| Mean (SD) | | | | | 39.714 (3.129) |
| % Coefficient of Variation (CV) | | | | | 7.88 |
| P-value for Wilcoxon’s Paired Test | | | | | 0.057 |

* PGS range based on the number of genotyped SNPs available on the GSA for each family for PGS computation

**Supplementary Table 11**. PW-PGS [NumSNPs=39]

| FamID | Num SNPs available  [PGS range*] | PGS based on cord blood genotype | PGS based on prediction | Difference between cord blood and predicted PGS | Mean PGS –  Cord blood and predicted |
| --- | --- | --- | --- | --- | --- |
| FPS001M | 25 [0 - 50] | 37 | 38 | -1 | 37.5 |
| FPS002M | 24 [0 - 48] | 35 | 34 | 1 | 34.5 |
| FPS003M | 25 [0 - 50] | 32 | 34 | -2 | 33 |
| FPS005M | 25 [0 - 50] | 31 | 34 | -3 | 32.5 |
| FPS006M | 25 [0 - 50] | 37 | 38 | -1 | 37.5 |
| FPS008M | 23 [0 - 46] | 31 | 34 | -3 | 32.5 |
| FPS009M | 25 [0 - 50] | 36 | 35 | 1 | 35.5 |
| FPS011M | 25 [0 - 50] | 38 | 39 | -1 | 38.5 |
| FPS012M | 25 [0 - 50] | 34 | 33 | 1 | 33.5 |
| FPS013M | 24 [0 - 48] | 36 | 35 | 1 | 35.5 |
| FPS014M | 25 [0 - 50] | 40 | 38 | 2 | 39 |
| FPS015M | 25 [0 - 50] | 37 | 38 | -1 | 37.5 |
| FPS016M | 24 [0 - 48] | 34 | 32 | 2 | 33 |
| FPS019M | 25 [0 - 50] | 36 | 36 | 0 | 36 |
| FPS022M | 25 [0 - 50] | 37 | 35 | 2 | 36 |
| FPS025M | 25 [0 - 50] | 36 | 37 | -1 | 36.5 |
| FPS026M | 24 [0 - 48] | 35 | 36 | -1 | 35.5 |
| FPS027M | 25 [0 - 50] | 34 | 37 | -3 | 35.5 |
| FPS028M | 25 [0 - 50] | 40 | 35 | 5 | 37.5 |
| FPS029M | 24 [0 - 48] | 32 | 30 | 2 | 31 |
| FPS030M | 25 [0 - 50] | 31 | 35 | -4 | 33 |
| Mean (SD) | | | | | 35.286 (2.234) |
| % Coefficient of Variation (CV) | | | | | 6.33 |
| P-value for Wilcoxon Paired Test | | | | | 0.690 |

* PGS range based on the number of genotyped SNPs available on the GSA for each family for PGS computation

**Supplementary Table 12.** ADHD-PGS [NumSNPs = 27]

| FamID | Num SNPs available  [PGS range*] | PGS based on cord blood genotype | PGS based on prediction | Difference between cord blood and predicted PGS | Mean PGS –  Cord blood and predicted |
| --- | --- | --- | --- | --- | --- |
| FPS001M | 17 [0 - 34] | 18 | 21 | -3 | 19.5 |
| FPS002M | 18 [0 - 36] | 24 | 22 | 2 | 23 |
| FPS003M | 18 [0 - 36] | 17 | 21 | -4 | 19 |
| FPS005M | 18 [0 - 36] | 21 | 20 | 1 | 20.5 |
| FPS006M | 18 [0 - 36] | 22 | 22 | 0 | 22 |
| FPS008M | 17 [0 - 34] | 22 | 25 | -3 | 23.5 |
| FPS009M | 17 [0 - 34] | 18 | 16 | 2 | 17 |
| FPS011M | 17 [0 - 34] | 22 | 25 | -3 | 23.5 |
| FPS012M | 18 [0 - 36] | 19 | 19 | 0 | 19 |
| FPS013M | 17 [0 - 34] | 18 | 19 | -1 | 18.5 |
| FPS014M | 18 [0 - 36] | 21 | 21 | 0 | 21 |
| FPS015M | 17 [0 - 34] | 21 | 23 | -2 | 22 |
| FPS016M | 18 [0 - 36] | 16 | 18 | -2 | 17 |
| FPS019M | 18 [0 - 36] | 21 | 18 | 3 | 19.5 |
| FPS022M | 18 [0 - 36] | 16 | 15 | 1 | 15.5 |
| FPS025M | 16 [0 - 32] | 18 | 20 | -2 | 19 |
| FPS026M | 18 [0 - 36] | 21 | 21 | 0 | 21 |
| FPS027M | 17 [0 - 34] | 19 | 16 | 3 | 17.5 |
| FPS028M | 18 [0 - 36] | 25 | 21 | 4 | 23 |
| FPS029M | 17 [0 - 34] | 20 | 24 | -4 | 22 |
| FPS030M | 16 [0 - 32] | 16 | 14 | 2 | 15 |
| Mean (SD) | | | | | 19.905 (2.567) |
| % Coefficient of Variation (CV) | | | | | 12.90 |
| P-value for Wilcoxon’s Paired Test | | | | | 0.551 |

* PGS range based on the number of genotyped SNPs available on the GSA for each family for PGS computation

**Supplementary Table 13**. ChOB-PGS [NumSNPs=22]

| FamID | Num SNPs available  [PGS range*] | PGS based on cord blood genotype | PGS based on prediction | Difference between cord blood and predicted PGS | Mean PGS –  Cord blood and predicted |
| --- | --- | --- | --- | --- | --- |
| FPS001M | 13 [0 - 26] | 17 | 16 | 1 | 16.5 |
| FPS002M | 11 [0 - 22] | 8 | 10 | -2 | 9 |
| FPS003M | 13 [0 - 26] | 15 | 13 | 2 | 14 |
| FPS005M | 13 [0 - 26] | 15 | 13 | 2 | 14 |
| FPS006M | 13 [0 - 26] | 17 | 18 | -1 | 17.5 |
| FPS008M | 13 [0 - 26] | 18 | 14 | 4 | 16 |
| FPS009M | 13 [0 - 26] | 15 | 13 | 2 | 14 |
| FPS011M | 11 [0 - 22] | 12 | 13 | -1 | 12.5 |
| FPS012M | 13 [0 - 26] | 15 | 14 | 1 | 14.5 |
| FPS013M | 11 [0 - 22] | 12 | 13 | -1 | 12.5 |
| FPS014M | 13 [0 - 26] | 19 | 15 | 4 | 17 |
| FPS015M | 13 [0 - 26] | 10 | 11 | -1 | 10.5 |
| FPS016M | 12 [0 - 24] | 16 | 12 | 4 | 14 |
| FPS019M | 13 [0 - 26] | 17 | 19 | -2 | 18 |
| FPS022M | 13 [0 - 26] | 18 | 15 | 3 | 16.5 |
| FPS025M | 13 [0 - 26] | 15 | 15 | 0 | 15 |
| FPS026M | 12 [0 - 24] | 15 | 16 | -1 | 15.5 |
| FPS027M | 13 [0 - 26] | 17 | 19 | -2 | 18 |
| FPS028M | 11 [0 - 22] | 12 | 13 | -1 | 12.5 |
| FPS029M | 13 [0 - 26] | 16 | 11 | 5 | 13.5 |
| FPS030M | 12 [0 - 24] | 14 | 12 | 2 | 13 |
| Mean (SD) | | | | | 14.476 (2.385) |
| % Coefficient of Variation (CV) | | | | | 16.473 |
| P-value for Wilcoxon’s Paired Test | | | | | 0.117 |

* PGS range based on the number of genotyped SNPs available on the GSA for each family for PGS computation

**Supplementary Table 14.** Overall mean and standard deviation for (percent) prediction accuracy of all SNPs (Fetal SNPclasses F1 to F9) predicted with the use of certainty method [F1, F3, F7 and F9] and 90% CI for maternal SNPs SIP [“BAF” for SNPs in Fetal SNPclasses F2, F4, and F6; SIP “NormR” for SNPs in Fetal SNPclass F5; SIP “θ” for SNPs in Fetal SNPclass F8]

| FamID | A/C SNPs | A/G SNPs | C/T SNPs | T/G SNPs | All  Base-Pairings |
| --- | --- | --- | --- | --- | --- |
| FPS001M | 86.13 | 86.56 | 86.39 | 86.09 | 86.41 |
| FPS002M | 86.52 | 86.79 | 86.83 | 86.32 | 86.74 |
| FPS003M | 86.83 | 87.16 | 87.03 | 86.65 | 87.03 |
| FPS005M | 85.27 | 85.73 | 85.67 | 85.20 | 85.61 |
| FPS006M | 86.83 | 87.30 | 87.16 | 86.80 | 87.15 |
| FPS008M | 85.93 | 86.14 | 86.01 | 86.02 | 86.06 |
| FPS009M | 85.10 | 85.40 | 85.29 | 85.16 | 85.30 |
| FPS011M | 87.17 | 87.66 | 87.46 | 87.09 | 87.48 |
| FPS012M | 86.87 | 87.54 | 87.42 | 87.13 | 87.39 |
| FPS013M | 85.82 | 86.32 | 86.38 | 85.83 | 86.25 |
| FPS014M | 86.91 | 87.28 | 87.19 | 86.75 | 87.16 |
| FPS015M | 87.52 | 88.14 | 87.88 | 87.62 | 87.93 |
| FPS016M | 86.00 | 86.70 | 86.54 | 86.31 | 86.53 |
| FPS019M | 87.22 | 87.84 | 87.61 | 87.20 | 87.63 |
| FPS022M | 84.97 | 85.62 | 85.44 | 85.04 | 85.43 |
| FPS025M | 85.05 | 85.50 | 85.23 | 84.83 | 85.28 |
| FPS026M | 84.46 | 85.04 | 84.70 | 84.41 | 84.79 |
| FPS027M | 85.20 | 85.56 | 85.58 | 85.18 | 85.50 |
| FPS028M | 84.50 | 84.69 | 84.59 | 84.36 | 84.60 |
| FPS029M | 86.69 | 86.91 | 86.74 | 86.44 | 86.78 |
| FPS030M | 86.59 | 87.32 | 87.15 | 86.88 | 87.14 |

**Supplementary Table 15.** Mean and standard deviation for (percent) prediction accuracy across all study families for SNPs in SNPclasses F2, F4, F5, F6 and F8 (all SNP base-pairing) predicted 90% CI for maternal SNPs

| SIP | F2 | F4 | F5 | F6 | F8 | Overall Accuracy |
| --- | --- | --- | --- | --- | --- | --- |
|  | Mean (SD) | Mean (SD) | Mean (SD) | Mean (SD) | Mean (SD) | Mean (SD) |
| RawX | 49.98 (0.52) | 50.03 (0.44) | 25.28 (0.54) | 50.52 (0.49) | 53.50 (2.67) | 45.56 (0.76) |
| RawY | 55.30 (3.75) | 50.45 (0.36) | 25.51 (0.62) | 50.20 (0.40) | 49.99 (0.47) | 45.65 (0.89) |
| NormX | 50.11 (0.41) | 50.45 (0.44) | 25.53 (0.67) | 50.58 (0.48) | 56.70 (4.76) | 46.40 (1.31) |
| NormY | 58.22 (5.21) | 50.59 (0.36) | 25.64 (0.70) | 50.41 (0.50) | 50.14 (0.46) | 46.52 (1.27) |
| NormR | 50.11 (0.32) | 49.90 (0.27) | 35.84 (1.39) | 50.11 (0.35) | 50.14 (0.33) | 47.02 (0.34) |
| θ | 56.63 (5.12) | 52.41 (1.49) | 27.56 (1.71) | 52.39 (1.48) | 56.99 (5.54) | 48.83 (3.01) |
| BAF | 58.42 (4.97) | 53.55 (2.23) | 28.83 (2.60) | 53.86 (2.64) | 53.70 (2.36) | 49.26 (2.78) |
| Best Predictor | BAF | BAF | NormR | BAF | θ |  |

**Supplementary Table 16.** Mean and standard deviation for (percent) prediction accuracy across all study families for A/C SNPs in SNPclasses F2, F4, F5, F6 and F8 predicted with 90% CI for maternal SNPs

| SIP | F2 | F4 | F5 | F6 | F8 | Overall Accuracy |
| --- | --- | --- | --- | --- | --- | --- |
|  | Mean (SD) | Mean (SD) | Mean (SD) | Mean (SD) | Mean (SD) | Mean (SD) |
| RawX | 50.15 (0.82) | 50.01 (0.88) | 25.42 (0.83) | 50.88 (1.01) | 53.17 (2.51) | 45.55 (0.82) |
| RawY | 55.35 (3.55) | 50.21 (0.73) | 25.74 (0.96) | 50.35 (0.76) | 50.21 (0.87) | 45.92 (0.78) |
| NormX | 50.16 (0.84) | 50.16 (1.12) | 25.76 (1.01) | 50.90 (0.89) | 56.56 (4.83) | 46.35 (1.45) |
| NormY | 58.06 (5.08) | 50.31 (1.04) | 25.95 (1.08) | 50.53 (0.88) | 50.11 (1.05) | 46.50 (1.22) |
| NormR | 50.21 (0.79) | 50.00 (1.01) | 35.62 (1.52) | 50.21 (0.96) | 50.09 (0.86) | 46.99 (0.56) |
| θ | 56.20 (5.02) | 51.86 (1.32) | 27.54 (1.62) | 52.25 (1.12) | 56.93 (5.64) | 48.54 (2.77) |
| BAF | 58.16 (4.92) | 53.52 (2.24) | 29.14 (2.57) | 53.93 (2.56) | 53.52 (2.22) | 49.21 (2.73) |
| Best Predictor | BAF | BAF | NormR | BAF | θ |  |

**Supplementary Table 17.** Mean and standard deviation for (percent) prediction accuracy across all study families for A/G SNPs in SNPclasses F2, F4, F5, F6 and F8 predicted with 90% CI for maternal SNPs

| SIP | F2 | F4 | F5 | F6 | F8 | Overall Accuracy |
| --- | --- | --- | --- | --- | --- | --- |
|  | Mean (SD) | Mean (SD) | Mean (SD) | Mean (SD) | Mean (SD) | Mean (SD) |
| RawX | 50.11 (0.75) | 49.89 (0.54) | 25.20 (0.52) | 50.50 (0.55) | 53.53 (2.72) | 45.58 (0.74) |
| RawY | 55.44 (3.80) | 50.63 (0.46) | 25.43 (0.74) | 50.23 (0.51) | 49.81 (0.63) | 45.89 (0.96) |
| NormX | 50.11 (0.59) | 40.47 (0.64) | 25.53 (0.69) | 50.48 (0.56) | 56.91 (4.87) | 46.46 (1.33) |
| NormY | 58.24 (5.18) | 50.67 (0.52) | 25.53 (0.78) | 50.42 (0.69) | 50.10 (0.59) | 46.53 (1.27) |
| NormR | 50.10 (0.59) | 49.88 (0.55) | 35.97 (1.39) | 50.09 (0.53) | 50.23 (0.40) | 47.07 (0.34) |
| θ | 56.71 (5.14) | 52.70 (1.63) | 27.64 (1.82) | 52.42 (1.60) | 57.07 (5.61) | 48.97 (3.10) |
| BAF | 58.75 (5.11) | 53.69 (2.33) | 28.82 (2.66) | 53.88 (2.72) | 53.84 (2.43) | 49.39 (2.85) |
| Best Predictor | BAF | BAF | NormR | BAF | θ |  |

**Supplementary Table 18.** Mean and standard deviation for (percent) prediction accuracy across all study families for T/C SNPs in SNPclasses F2, F4, F5, F6 and F8 predicted with 90% CI for maternal SNPs

| SIP | F2 | F4 | F5 | F6 | F8 | Overall Accuracy |
| --- | --- | --- | --- | --- | --- | --- |
|  | Mean (SD) | Mean (SD) | Mean (SD) | Mean (SD) | Mean (SD) | Mean (SD) |
| RawX | 49.86 (0.51) | 50.09 (0.59) | 25.35 (0.68) | 50.50 (0.61) | 53.54 (2.73) | 45.57 (0.81) |
| RawY | 55.22 (3.80) | 50.47 (0.54) | 25.46 (0.56) | 50.18 (0.46) | 50.05 (0.50) | 45.83 (0.86) |
| NormX | 50.09 (0.49) | 50.42 (0.41) | 25.51 (0.72) | 50.52 (0.57) | 56.56 (4.72) | 46.35 (1.30) |
| NormY | 58.22 (5.24) | 50.68 (0.50) | 25.64 (0.59) | 50.43 (0.55) | 50.18 (0.52) | 46.53 (1.28) |
| NormR | 50.11 (0.34) | 50.02 (0.35) | 35.78 (1.41) | 50.04 (0.45) | 50.09 (0.35) | 47.01 (0.35) |
| θ | 56.67 (5.18) | 52.37 (1.50) | 27.53 (1.71) | 52.49 (1.60) | 56.99 (5.53) | 48.84 (3.03) |
| BAF | 58.13 (4.82) | 53.45 (2.16) | 28.79 (2.56) | 53.91 (2.64) | 53.66 (2.36) | 49.17 (2.72) |
| Best Predictor | NormY | BAF | NormR | BAF | θ |  |

**Supplementary Table 19.** Mean and standard deviation for (percent) prediction accuracy across all study families for T/G SNPs in SNPclasses F2, F4, F5, F6 and F8 predicted with 90% CI for maternal SNPs

| SIP | F2 | F4 | F5 | F6 | F8 | Overall Accuracy |
| --- | --- | --- | --- | --- | --- | --- |
|  | Mean (SD) | Mean (SD) | Mean (SD) | Mean (SD) | Mean (SD) | Mean (SD) |
| RawX | 49.75 (0.90) | 50.39 (0.90) | 25.22 (0.82) | 50.36 (1.09) | 53.52 (2.76) | 45.48 (0.76) |
| RawY | 54.99 (3.74) | 49.87 (0.76) | 25.80 (1.01) | 49.95 (0.81) | 50.24 (1.02) | 45.73 (0.99) |
| NormX | 50.13 (0.91) | 50.80 (0.77) | 25.37 (1.13) | 50.96 (0.97) | 56.54 (4.63) | 46.39 (1.27) |
| NormY | 58.30 (5.50) | 50.22 (0.59) | 25.82 (1.13) | 50.20 (1.03) | 50.16 (0.70) | 46.46 (1.40) |
| NormR | 50.03 (0.92) | 49.35 (0.66) | 35.73 (1.70) | 50.38 (0.81) | 50.02 (0.88) | 46.89 (0.53) |
| θ | 56.57 (5.11) | 51.90 (1.63) | 27.38 (1.85) | 51.98 (1.58) | 56.66 (5.36) | 48.48 (2.85) |
| BAF | 58.52 (5.31) | 53.36 (2.40) | 28.77 (2.79) | 53.51 (2.70) | 53.53 (2.55) | 49.10 (2.83) |
| Best Predictor | BAF | BAF | NormR | BAF | θ |  |

**Supplementary Tables 20-25** present the comparison of predicted PGS (based on genotype call derived from the certainty and maternal 90% CI SNPs) and PGS based on cord blood. The number of SNPs identified in the published literature for each phenotype is listed before the start of each table.

**Supplementary Table 20**. BW-PGS2019 [NumSNPs=146]

| FamID | Num SNPs available  [PGS range*] | PGS based on cord blood genotype | PGS based on prediction | Difference between cord blood and predicted PGS | Mean PGS –  Cord blood and predicted |
| --- | --- | --- | --- | --- | --- |
| FPS001M | 74 [0 - 148] | 88 | 91 | -3 | 89.5 |
| FPS002M | 75 [0 - 150] | 86 | 81 | 5 | 83.5 |
| FPS003M | 76 [0 - 152] | 76 | 80 | -4 | 78 |
| FPS005M | 76 [0 - 152] | 91 | 86 | 5 | 88.5 |
| FPS006M | 76 [0 - 152] | 83 | 86 | -3 | 84.5 |
| FPS008M | 74 [0 - 148] | 81 | 83 | -2 | 82 |
| FPS009M | 75 [0 - 150] | 79 | 86 | -7 | 82.5 |
| FPS011M | 76 [0 - 152] | 90 | 87 | 3 | 88.5 |
| FPS012M | 75 [0 - 150] | 92 | 82 | 10 | 87 |
| FPS013M | 75 [0 - 150] | 86 | 76 | 10 | 81 |
| FPS014M | 76 [0 - 152] | 97 | 100 | -3 | 98.5 |
| FPS015M | 76 [0 - 152] | 81 | 85 | -4 | 83 |
| FPS016M | 75 [0 - 150] | 84 | 87 | -3 | 85.5 |
| FPS019M | 76 [0 - 152] | 83 | 86 | -3 | 84.5 |
| FPS022M | 76 [0 - 152] | 94 | 96 | -2 | 95 |
| FPS025M | 75 [0 - 150] | 88 | 94 | -6 | 91 |
| FPS026M | 75 [0 - 150] | 100 | 87 | 13 | 93.5 |
| FPS027M | 72 [0 - 144] | 87 | 86 | 1 | 86.5 |
| FPS028M | 73 [0 - 146] | 82 | 91 | -9 | 86.5 |
| FPS029M | 75 [0 - 150] | 85 | 92 | -7 | 88.5 |
| FPS030M | 75 [0 - 150] | 93 | 84 | 9 | 88.5 |
| Mean (SD) | | | | | 86.952 (4.876) |
| % Coefficient of Variation (CV) | | | | | 5.61 |
| P-value for Wilcoxon’s Paired Test | | | | | 0.862 |

*PGS range based on the number of genotyped SNPs available on the GSA for each family for PGS computation

**Supplementary Table 21**. Dep-PGS [NumSNPs=102]

| FamID | Num SNPs available  [PGS range*] | PGS based on cord blood genotype | PGS based on prediction | Difference between cord blood and predicted PGS | Mean PGS –  Cord blood and predicted |
| --- | --- | --- | --- | --- | --- |
| FPS001M | 63 [0 - 126] | 72 | 69 | 3 | 70.5 |
| FPS002M | 60 [0 - 120] | 68 | 64 | 4 | 66 |
| FPS003M | 63 [0 - 126] | 74 | 71 | 3 | 72.5 |
| FPS005M | 63 [0 - 126] | 62 | 64 | -2 | 63 |
| FPS006M | 63 [0 - 126] | 78 | 72 | 6 | 75 |
| FPS008M | 59 [0 - 118] | 63 | 57 | 6 | 60 |
| FPS009M | 62 [0 - 124] | 69 | 71 | -2 | 70 |
| FPS011M | 62 [0 - 124] | 67 | 70 | -3 | 68.5 |
| FPS012M | 63 [0 - 126] | 67 | 67 | 0 | 67 |
| FPS013M | 62 [0 - 124] | 63 | 60 | 3 | 61.5 |
| FPS014M | 62 [0 - 124] | 65 | 59 | 6 | 62 |
| FPS015M | 63 [0 - 126] | 76 | 80 | -4 | 78 |
| FPS016M | 62 [0 - 124] | 64 | 62 | 2 | 63 |
| FPS019M | 63 [0 - 126] | 67 | 72 | -5 | 69.5 |
| FPS022M | 63 [0 - 126] | 67 | 65 | 2 | 66 |
| FPS025M | 63 [0 - 126] | 67 | 69 | -2 | 68 |
| FPS026M | 62 [0 - 124] | 65 | 70 | -5 | 67.5 |
| FPS027M | 63 [0 - 126] | 70 | 72 | -2 | 71 |
| FPS028M | 60 [0 - 120] | 72 | 64 | 8 | 68 |
| FPS029M | 62 [0 - 124] | 64 | 59 | 5 | 61.5 |
| FPS030M | 62 [0 - 124] | 71 | 73 | -2 | 72 |
| Mean (SD) | | | | | 67.643 (4.733) |
| % Coefficient of Variation (CV) | | | | | 7.00 |
| P-value for Wilcoxon Paired Test | | | | | 0.222 |

* PGS range based on the number of genotyped SNPs available on the GSA for each family for PGS computation

**Supplementary Table 22**. BW-PGS2016 [NumSNPs=60]

| FamID | Num SNPs available  [PGS range*] | PGS based on cord blood genotype | PGS based on prediction | Difference between cord blood and predicted PGS | Mean PGS –  Cord blood and predicted |
| --- | --- | --- | --- | --- | --- |
| FPS001M | 31 [0 - 62] | 43 | 40 | 3 | 41.5 |
| FPS002M | 31 [0 - 62] | 38 | 34 | 4 | 36 |
| FPS003M | 32 [0 - 64] | 38 | 39 | -1 | 38.5 |
| FPS005M | 32 [0 - 64] | 41 | 43 | -2 | 42 |
| FPS006M | 32 [0 - 64] | 38 | 32 | 6 | 35 |
| FPS008M | 32 [0 - 64] | 39 | 41 | -2 | 40 |
| FPS009M | 32 [0 - 64] | 34 | 35 | -1 | 34.5 |
| FPS011M | 32 [0 - 64] | 41 | 35 | 6 | 38 |
| FPS012M | 31 [0 - 62] | 37 | 38 | -1 | 37.5 |
| FPS013M | 31 [0 - 62] | 38 | 37 | 1 | 37.5 |
| FPS014M | 32 [0 - 64] | 41 | 43 | -2 | 42 |
| FPS015M | 32 [0 - 64] | 42 | 39 | 3 | 40.5 |
| FPS016M | 31 [0 - 62] | 36 | 32 | 4 | 34 |
| FPS019M | 32 [0 - 64] | 42 | 37 | 5 | 39.5 |
| FPS022M | 32 [0 - 64] | 44 | 44 | 0 | 44 |
| FPS025M | 32 [0 - 64] | 39 | 40 | -1 | 39.5 |
| FPS026M | 31 [0 - 62] | 41 | 41 | 0 | 41 |
| FPS027M | 32 [0 - 64] | 45 | 45 | 0 | 45 |
| FPS028M | 32 [0 - 64] | 43 | 40 | 3 | 41.5 |
| FPS029M | 32 [0 - 64] | 43 | 44 | -1 | 43.5 |
| FPS030M | 32 [0 - 64] | 45 | 40 | 5 | 42.5 |
| Mean (SD) | | | | | 39.690 (3.144) |
| % Coefficient of Variation (CV) | | | | | 7.92 |
| P-value for Wilcoxon’s Paired Test | | | | | 0.057 |

* PGS range based on the number of genotyped SNPs available on the GSA for each family for PGS computation

**Supplementary Table 23.** PW-PGS [NumSNPs=39]

| FamID | Num SNPs available  [PGS range*] | PGS based on cord blood genotype | PGS based on prediction | Difference between cord blood and predicted PGS | Mean PGS –  Cord blood and predicted |
| --- | --- | --- | --- | --- | --- |
| FPS001M | 25 [0 - 50] | 37 | 38 | -1 | 37.5 |
| FPS002M | 24 [0 - 48] | 35 | 34 | 1 | 34.5 |
| FPS003M | 25 [0 - 50] | 32 | 34 | -2 | 33 |
| FPS005M | 25 [0 - 50] | 31 | 34 | -3 | 32.5 |
| FPS006M | 25 [0 - 50] | 37 | 38 | -1 | 37.5 |
| FPS008M | 23 [0 - 46] | 31 | 34 | -3 | 32.5 |
| FPS009M | 25 [0 - 50] | 36 | 35 | 1 | 35.5 |
| FPS011M | 25 [0 - 50] | 38 | 39 | -1 | 38.5 |
| FPS012M | 25 [0 - 50] | 34 | 33 | 1 | 33.5 |
| FPS013M | 24 [0 - 48] | 36 | 35 | 1 | 35.5 |
| FPS014M | 25 [0 - 50] | 40 | 38 | 2 | 39 |
| FPS015M | 25 [0 - 50] | 37 | 38 | -1 | 37.5 |
| FPS016M | 24 [0 - 48] | 34 | 32 | 2 | 33 |
| FPS019M | 25 [0 - 50] | 36 | 36 | 0 | 36 |
| FPS022M | 25 [0 - 50] | 37 | 35 | 2 | 36 |
| FPS025M | 25 [0 - 50] | 36 | 37 | -1 | 36.5 |
| FPS026M | 24 [0 - 48] | 35 | 36 | -1 | 35.5 |
| FPS027M | 25 [0 - 50] | 34 | 37 | -3 | 35.5 |
| FPS028M | 25 [0 - 50] | 40 | 33 | 7 | 36.5 |
| FPS029M | 24 [0 - 48] | 32 | 30 | 2 | 31 |
| FPS030M | 25 [0 - 50] | 31 | 35 | -4 | 33 |
| Mean (SD) | | | | | 35.238 (2.194) |
| % Coefficient of Variation (CV) | | | | | 6.23 |
| P-value for Wilcoxon Paired Test | | | | | 0.690 |

* PGS range based on the number of genotyped SNPs available on the GSA for each family for PGS computation

**Supplementary Table 24.** ADHD-PGS [NumSNPs = 27]

| FamID | Num SNPs available  [PGS range*] | PGS based on cord blood genotype | PGS based on prediction | Difference between cord blood and predicted PGS | Mean PGS –  Cord blood and predicted |
| --- | --- | --- | --- | --- | --- |
| FPS001M | 17 [0 - 34] | 18 | 21 | -3 | 19.5 |
| FPS002M | 18 [0 - 36] | 24 | 22 | 2 | 23 |
| FPS003M | 18 [0 - 36] | 17 | 22 | -5 | 19.5 |
| FPS005M | 18 [0 - 36] | 21 | 20 | 1 | 20.5 |
| FPS006M | 18 [0 - 36] | 22 | 22 | 0 | 22 |
| FPS008M | 17 [0 - 34] | 22 | 25 | -3 | 23.5 |
| FPS009M | 17 [0 - 34] | 18 | 16 | 2 | 17 |
| FPS011M | 17 [0 - 34] | 22 | 25 | -3 | 23.5 |
| FPS012M | 18 [0 - 36] | 19 | 19 | 0 | 19 |
| FPS013M | 17 [0 - 34] | 18 | 19 | -1 | 18.5 |
| FPS014M | 18 [0 - 36] | 21 | 21 | 0 | 21 |
| FPS015M | 17 [0 - 34] | 21 | 23 | -2 | 22 |
| FPS016M | 18 [0 - 36] | 16 | 18 | -2 | 17 |
| FPS019M | 18 [0 - 36] | 21 | 18 | 3 | 19.5 |
| FPS022M | 18 [0 - 36] | 16 | 15 | 1 | 15.5 |
| FPS025M | 16 [0 - 32] | 18 | 20 | -2 | 19 |
| FPS026M | 18 [0 - 36] | 21 | 21 | 0 | 21 |
| FPS027M | 17 [0 - 34] | 19 | 16 | 3 | 17.5 |
| FPS028M | 18 [0 - 36] | 25 | 21 | 4 | 23 |
| FPS029M | 17 [0 - 34] | 20 | 24 | -4 | 22 |
| FPS030M | 16 [0 - 32] | 16 | 14 | 2 | 15 |
| Mean (SD) | | | | | 19.929 (2.561) |
| % Coefficient of Variation (CV) | | | | | 12.85 |
| P-value for Wilcoxon’s Paired Test | | | | | 0.535 |

* PGS range based on the number of genotyped SNPs available on the GSA for each family for PGS computation

**Supplementary Table 25.** ChOB-PGS [NumSNPs=22]

| FamID | Num SNPs available  [PGS range*] | PGS based on cord blood genotype | PGS based on prediction | Difference between cord blood and predicted PGS | Mean PGS –  Cord blood and predicted |
| --- | --- | --- | --- | --- | --- |
| FPS001M | 13 [0 - 26] | 17 | 16 | 1 | 16.5 |
| FPS002M | 11 [0 - 22] | 8 | 10 | -2 | 9 |
| FPS003M | 13 [0 - 26] | 15 | 13 | 2 | 14 |
| FPS005M | 13 [0 - 26] | 15 | 13 | 2 | 14 |
| FPS006M | 13 [0 - 26] | 17 | 18 | -1 | 17.5 |
| FPS008M | 13 [0 - 26] | 18 | 14 | 4 | 16 |
| FPS009M | 13 [0 - 26] | 15 | 13 | 2 | 14 |
| FPS011M | 11 [0 - 22] | 12 | 13 | -1 | 12.5 |
| FPS012M | 13 [0 - 26] | 15 | 14 | 1 | 14.5 |
| FPS013M | 11 [0 - 22] | 12 | 13 | -1 | 12.5 |
| FPS014M | 13 [0 - 26] | 19 | 15 | 4 | 17 |
| FPS015M | 13 [0 - 26] | 10 | 11 | -1 | 10.5 |
| FPS016M | 12 [0 - 24] | 16 | 12 | 4 | 14 |
| FPS019M | 13 [0 - 26] | 17 | 19 | -2 | 18 |
| FPS022M | 13 [0 - 26] | 18 | 15 | 3 | 16.5 |
| FPS025M | 13 [0 - 26] | 15 | 15 | 0 | 15 |
| FPS026M | 12 [0 - 24] | 15 | 16 | -1 | 15.5 |
| FPS027M | 13 [0 - 26] | 17 | 19 | -2 | 18 |
| FPS028M | 11 [0 - 22] | 12 | 13 | -1 | 12.5 |
| FPS029M | 13 [0 - 26] | 16 | 11 | 5 | 13.5 |
| FPS030M | 12 [0 - 24] | 14 | 12 | 2 | 13 |
| Mean (SD) | | | | | 14.476 (2.385) |
| % Coefficient of Variation (CV) | | | | | 16.473 |
| P-value for Wilcoxon’s Paired Test | | | | | 0.117 |

* PGS range based on the number of genotyped SNPs available on the GSA for each family for PGS computation

**Supplementary Table 26.** Overall mean and standard deviation for (percent) prediction accuracy of all SNPs (Fetal SNPclasses F1 to F9) predicted with the use of certainty method [F1, F3, F7 and F9] and 99% CI for maternal SNPs SIP [“BAF” for SNPs in Fetal SNPclasses F2, F4, and F6; SIP “NormR” for SNPs in Fetal SNPclass F5; SIP “θ” for SNPs in Fetal SNPclass F8]

| FamID | A/C SNPs | A/G SNPs | C/T SNPs | T/G SNPs | All  Base-Pairings |
| --- | --- | --- | --- | --- | --- |
| FPS001M | 86.14 | 86.56 | 86.39 | 86.10 | 86.41 |
| FPS002M | 86.52 | 86.80 | 86.83 | 86.32 | 86.74 |
| FPS003M | 86.83 | 87.16 | 87.03 | 86.67 | 87.03 |
| FPS005M | 85.30 | 85.73 | 85.66 | 85.20 | 85.61 |
| FPS006M | 86.84 | 87.30 | 87.16 | 86.79 | 87.15 |
| FPS008M | 85.93 | 86.14 | 86.01 | 86.02 | 86.06 |
| FPS009M | 85.10 | 85.40 | 85.29 | 85.16 | 85.30 |
| FPS011M | 87.18 | 87.67 | 87.46 | 87.10 | 87.48 |
| FPS012M | 86.88 | 87.54 | 87.43 | 87.14 | 87.40 |
| FPS013M | 85.80 | 86.32 | 86.38 | 85.83 | 86.25 |
| FPS014M | 86.91 | 87.28 | 87.19 | 86.76 | 87.16 |
| FPS015M | 87.53 | 88.14 | 87.89 | 87.62 | 87.93 |
| FPS016M | 86.00 | 86.70 | 86.54 | 86.32 | 86.53 |
| FPS019M | 87.21 | 87.84 | 87.62 | 87.21 | 87.63 |
| FPS022M | 84.97 | 85.62 | 85.44 | 85.05 | 85.43 |
| FPS025M | 85.04 | 85.50 | 85.23 | 84.83 | 85.28 |
| FPS026M | 84.46 | 85.04 | 84.70 | 84.41 | 84.79 |
| FPS027M | 85.19 | 85.56 | 85.59 | 85.17 | 85.50 |
| FPS028M | 84.51 | 84.69 | 84.59 | 84.36 | 84.60 |
| FPS029M | 86.70 | 86.91 | 86.74 | 86.44 | 86.78 |
| FPS030M | 86.60 | 87.32 | 87.15 | 86.87 | 87.14 |

**Supplementary Table 27.** Mean and standard deviation for (percent) prediction accuracy across all study families for SNPs in SNPclasses F2, F4, F5, F6 and F8 (all SNP base-pairing) predicted 99% CI for maternal SNPs

| SIP | F2 | F4 | F5 | F6 | F8 | Overall Accuracy |
| --- | --- | --- | --- | --- | --- | --- |
|  | Mean (SD) | Mean (SD) | Mean (SD) | Mean (SD) | Mean (SD) | Mean (SD) |
| RawX | 49.98 (0.52) | 50.03 (0.44) | 25.30 (0.54) | 50.53 (0.49) | 53.49 (2.67) | 45.57 (0.76) |
| RawY | 55.29 (3.75) | 50.45 (0.37) | 25.53 (0.62) | 50.20 (0.40) | 49.99 (0.48) | 45.86 (0.89) |
| NormX | 50.11 (0.41) | 50.45 (0.44) | 25.55 (0.67) | 50.58 (0.48) | 56.70 (4.77) | 46.41 (1.31) |
| NormY | 58.21 (5.21) | 50.59 (0.36) | 25.66 (0.70) | 50.42 (0.51) | 50.14 (0.46) | 46.52 (1.27) |
| NormR | 50.11 (0.32) | 49.89 (0.27) | 35.85 (1.39) | 50.10 (0.36) | 50.14 (0.33) | 47.02 (0.34) |
| θ | 56.62 (5.11) | 52.41 (1.49) | 27.59 (1.72) | 52.39 (1.48) | 56.99 (5.54) | 48.84 (3.01) |
| BAF | 58.44 (4.98) | 53.55 (2.23) | 28.85 (2.60) | 53.86 (2.64) | 53.71 (2.36) | 49.27 (2.78) |
| Best Predictor | BAF | BAF | NormR | BAF | θ |  |

**Supplementary Table 28.** Mean and standard deviation for (percent) prediction accuracy across all study families for A/C SNPs in SNPclasses F2, F4, F5, F6 and F8 predicted with 99% CI for maternal SNPs

| SIP | F2 | F4 | F5 | F6 | F8 | Overall Accuracy |
| --- | --- | --- | --- | --- | --- | --- |
|  | Mean (SD) | Mean (SD) | Mean (SD) | Mean (SD) | Mean (SD) | Mean (SD) |
| RawX | 50.16 (0.82) | 50.00 (0.87) | 25.47 (0.83) | 50.90 (1.01) | 53.15 (2.49) | 45.56 (0.82) |
| RawY | 55.34 (3.55) | 50.22 (0.74) | 25.79 (0.99) | 50.35 (0.76) | 50.21 (0.86) | 45.93 (0.77) |
| NormX | 50.16 (0.84) | 50.18 (1.12) | 25.80 (1.03) | 50.91 (0.91) | 56.55 (4.85) | 46.36 (1.47) |
| NormY | 58.06 (5.06) | 50.29 (1.05) | 25.98 (1.07) | 50.55 (0.87) | 50.12 (1.05) | 46.51 (1.21) |
| NormR | 50.21 (0.78) | 49.99 (1.00) | 35.65 (1.53) | 50.21 (0.96) | 50.09 (0.86) | 47.00 (0.56) |
| θ | 56.17 (5.00) | 51.89 (1.32) | 27.59 (1.63) | 52.25 (1.12) | 56.95 (5.66) | 48.55 (2.77) |
| BAF | 58.17 (4.92) | 53.54 (2.24) | 29.18 (2.56) | 53.91 (2.56) | 53.52 (2.21) | 49.22 (2.73) |
| Best Predictor | BAF | BAF | NormR | BAF | θ |  |

**Supplementary Table 29.** Mean and standard deviation for (percent) prediction accuracy across all study families for A/G SNPs in SNPclasses F2, F4, F5, F6 and F8 predicted with 99% CI for maternal SNPs

| SIP | F2 | F4 | F5 | F6 | F8 | Overall Accuracy |
| --- | --- | --- | --- | --- | --- | --- |
|  | Mean (SD) | Mean (SD) | Mean (SD) | Mean (SD) | Mean (SD) | Mean (SD) |
| RawX | 50.11 (0.75) | 49.89 (0.55) | 25.22 (0.52) | 50.50 (0.55) | 53.52 (2.71) | 45.58 (0.74) |
| RawY | 55.44 (3.80) | 50.63 (0.46) | 25.45 (0.74) | 50.23 (0.51) | 49.81 (0.63) | 45.89 (0.96) |
| NormX | 50.11 (0.59) | 50.47 (0.64) | 25.54 (0.69) | 50.48 (0.56) | 56.91 (4.88) | 46.47 (1.32) |
| NormY | 58.22 (5.18) | 50.67 (0.51) | 25.55 (0.78) | 50.42 (0.69) | 50.10 (0.58) | 46.53 (1.27) |
| NormR | 50.10 (0.59) | 49.88 (0.55) | 35.98 (1.39) | 50.08 (0.54) | 50.22 (0.41) | 47.07 (0.33) |
| θ | 56.71 (5.14) | 52.70 (1.62) | 27.66 (1.81) | 52.42 (1.60) | 57.06 (5.61) | 48.97 (3.10) |
| BAF | 58.77 (5.12) | 53.69 (2.33) | 28.82 (2.65) | 53.88 (2.72) | 53.84 (2.44) | 49.40 (2.85) |
| Best Predictor | BAF | BAF | NormR | BAF | θ |  |

**Supplementary Table 30.** Mean and standard deviation for (percent) prediction accuracy across all study families for T/C SNPs in SNPclasses F2, F4, F5, F6 and F8 predicted with 99% CI for maternal SNPs

| SIP | F2 | F4 | F5 | F6 | F8 | Overall Accuracy |
| --- | --- | --- | --- | --- | --- | --- |
|  | Mean (SD) | Mean (SD) | Mean (SD) | Mean (SD) | Mean (SD) | Mean (SD) |
| RawX | 49.86 (0.51) | 50.10 (0.59) | 25.36 (0.67) | 50.50 (0.61) | 53.54 (2.73) | 45.58 (0.81) |
| RawY | 55.22 (3.80) | 50.47 (0.54) | 25.48 (0.56) | 50.19 (0.45) | 50.05 (0.50) | 45.84 (0.86) |
| NormX | 50.09 (0.49) | 50.41 (0.41) | 25.53 (0.72) | 50.52 (0.56) | 56.56 (4.72) | 46.36 (1.30) |
| NormY | 58.21 (5.25) | 50.68 (0.51) | 25.66 (0.59) | 50.43 (0.55) | 50.18 (0.53) | 46.54 (1.28) |
| NormR | 50.11 (0.33) | 50.02 (0.35) | 35.79 (1.40) | 50.04 (0.44) | 50.09 (0.35) | 47.01 (0.35) |
| θ | 56.66 (5.18) | 52.38 (1.50) | 27.56 (1.72) | 52.49 (1.60) | 56.99 (5.53) | 48.85 (3.03) |
| BAF | 58.15 (4.83) | 53.46 (2.17) | 28.81 (2.57) | 53.90 (2.64) | 53.66 (2.37) | 49.18 (2.73) |
| Best Predictor | NormY | BAF | NormR | BAF | θ |  |

**Supplementary Table 31.** Mean and standard deviation for (percent) prediction accuracy across all study families for T/G SNPs in SNPclasses F2, F4, F5, F6 and F8 predicted with 99% CI for maternal SNPs

| SIP | F2 | F4 | F5 | F6 | F8 | Overall Accuracy |
| --- | --- | --- | --- | --- | --- | --- |
|  | Mean (SD) | Mean (SD) | Mean (SD) | Mean (SD) | Mean (SD) | Mean (SD) |
| RawX | 49.73 (0.89) | 50.37 (0.90) | 25.25 (0.81) | 50.36 (1.09) | 53.53 (2.76) | 45.48 (0.75) |
| RawY | 54.99 (3.75) | 49.87 (0.76) | 25.85 (1.01) | 49.95 (0.82) | 50.27 (1.02) | 45.74 (0.99) |
| NormX | 50.12 (0.90) | 50.81 (0.75) | 25.43 (1.13) | 50.95 (0.99) | 56.55 (4.64) | 46.41 (1.27) |
| NormY | 58.29 (5.51) | 50.22 (0.58) | 25.83 (1.12) | 50.18 (1.03) | 50.15 (0.70) | 46.45 (1.40) |
| NormR | 50.05 (0.92) | 49.33 (0.66) | 35.74 (1.71) | 50.38 (0.83) | 50.03 (0.88) | 46.89 (0.52) |
| θ | 56.55 (5.11) | 51.90 (1.64) | 27.44 (1.86) | 51.99 (1.60) | 56.67 (5.36) | 48.49 (2.85) |
| BAF | 58.55 (5.34) | 53.36 (2.41) | 28.81 (2.78) | 53.51 (2.69) | 53.55 (2.55) | 49.12 (2.83) |
| Best Predictor | BAF | BAF | NormR | BAF | θ |  |

**Supplementary Tables 32-37** present the comparison of predicted PGS (based on genotype call derived from the certainty and maternal 99% CI SNPs) and PGS based on cord blood. The number of SNPs identified in the published literature for each phenotype is listed before the start of each table.

**Supplementary Table 32.** BW-PGS2019 [NumSNPs=146]

| FamID | Num SNPs available  [PGS range*] | PGS based on cord blood genotype | PGS based on prediction | Difference between cord blood and predicted PGS | Mean PGS –  Cord blood and predicted |
| --- | --- | --- | --- | --- | --- |
| FPS001M | 74 [0 - 148] | 88 | 91 | -3 | 89.5 |
| FPS002M | 75 [0 - 150] | 86 | 80 | 6 | 83 |
| FPS003M | 76 [0 - 152] | 76 | 80 | -4 | 78 |
| FPS005M | 76 [0 - 152] | 91 | 86 | 5 | 88.5 |
| FPS006M | 76 [0 - 152] | 83 | 86 | -3 | 84.5 |
| FPS008M | 74 [0 - 148] | 81 | 83 | -2 | 82 |
| FPS009M | 75 [0 - 150] | 79 | 86 | -7 | 82.5 |
| FPS011M | 76 [0 - 152] | 90 | 87 | 3 | 88.5 |
| FPS012M | 75 [0 - 150] | 92 | 82 | 10 | 87 |
| FPS013M | 75 [0 - 150] | 86 | 77 | 9 | 81.5 |
| FPS014M | 76 [0 - 152] | 97 | 100 | -3 | 98.5 |
| FPS015M | 76 [0 - 152] | 81 | 85 | -4 | 83 |
| FPS016M | 75 [0 - 150] | 84 | 87 | -3 | 85.5 |
| FPS019M | 76 [0 - 152] | 83 | 86 | -3 | 84.5 |
| FPS022M | 76 [0 - 152] | 94 | 96 | -2 | 95 |
| FPS025M | 75 [0 - 150] | 88 | 94 | -6 | 91 |
| FPS026M | 75 [0 - 150] | 100 | 87 | 13 | 93.5 |
| FPS027M | 72 [0 - 144] | 87 | 86 | 1 | 86.5 |
| FPS028M | 73 [0 - 146] | 82 | 91 | -9 | 86.5 |
| FPS029M | 75 [0 - 150] | 85 | 92 | -7 | 88.5 |
| FPS030M | 75 [0 - 150] | 93 | 84 | 9 | 88.5 |
| Mean (SD) | | | | | 86.952 (4.865) |
| % Coefficient of Variation (CV) | | | | | 5.60 |
| P-value for Wilcoxon’s Paired Test | | | | | 0.862 |

*PGS range based on the number of genotyped SNPs available on the GSA for each family for PGS computation

**Supplementary Table 33**. Dep-PGS [NumSNPs=102]

| FamID | Num SNPs available  [PGS range*] | PGS based on cord blood genotype | PGS based on prediction | Difference between cord blood and predicted PGS | Mean PGS –  Cord blood and predicted |
| --- | --- | --- | --- | --- | --- |
| FPS001M | 63 [0 - 126] | 72 | 69 | 3 | 70.5 |
| FPS002M | 60 [0 - 120] | 68 | 64 | 4 | 66 |
| FPS003M | 63 [0 - 126] | 74 | 71 | 3 | 72.5 |
| FPS005M | 63 [0 - 126] | 62 | 64 | -2 | 63 |
| FPS006M | 63 [0 - 126] | 78 | 72 | 6 | 75 |
| FPS008M | 59 [0 - 118] | 63 | 57 | 6 | 60 |
| FPS009M | 62 [0 - 124] | 69 | 71 | -2 | 70 |
| FPS011M | 62 [0 - 124] | 67 | 70 | -3 | 68.5 |
| FPS012M | 63 [0 - 126] | 67 | 67 | 0 | 67 |
| FPS013M | 62 [0 - 124] | 63 | 60 | 3 | 61.5 |
| FPS014M | 62 [0 - 124] | 65 | 59 | 6 | 62 |
| FPS015M | 63 [0 - 126] | 76 | 80 | -4 | 78 |
| FPS016M | 62 [0 - 124] | 64 | 62 | 2 | 63 |
| FPS019M | 63 [0 - 126] | 67 | 72 | -5 | 69.5 |
| FPS022M | 63 [0 - 126] | 67 | 65 | 2 | 66 |
| FPS025M | 63 [0 - 126] | 67 | 69 | -2 | 68 |
| FPS026M | 62 [0 - 124] | 65 | 70 | -5 | 67.5 |
| FPS027M | 63 [0 - 126] | 70 | 72 | -2 | 71 |
| FPS028M | 60 [0 - 120] | 72 | 64 | 8 | 68 |
| FPS029M | 62 [0 - 124] | 64 | 59 | 5 | 61.5 |
| FPS030M | 62 [0 - 124] | 71 | 73 | -2 | 72 |
| Mean (SD) | | | | | 67.643 (4.733) |
| % Coefficient of Variation (CV) | | | | | 7.00 |
| P-value for Wilcoxon Paired Test | | | | | 0.222 |

* PGS range based on the number of genotyped SNPs available on the GSA for each family for PGS computation

**Supplementary Table 34**. BW-PGS2016 [NumSNPs=60]

| FamID | Num SNPs available  [PGS range*] | PGS based on cord blood genotype | PGS based on prediction | Difference between cord blood and predicted PGS | Mean PGS –  Cord blood and predicted |
| --- | --- | --- | --- | --- | --- |
| FPS001M | 31 [0 - 62] | 43 | 40 | 3 | 41.5 |
| FPS002M | 31 [0 - 62] | 38 | 34 | 4 | 36 |
| FPS003M | 32 [0 - 64] | 38 | 39 | -1 | 38.5 |
| FPS005M | 32 [0 - 64] | 41 | 43 | -2 | 42 |
| FPS006M | 32 [0 - 64] | 38 | 32 | 6 | 35 |
| FPS008M | 32 [0 - 64] | 39 | 41 | -2 | 40 |
| FPS009M | 32 [0 - 64] | 34 | 35 | -1 | 34.5 |
| FPS011M | 32 [0 - 64] | 41 | 35 | 6 | 38 |
| FPS012M | 31 [0 - 62] | 37 | 38 | -1 | 37.5 |
| FPS013M | 31 [0 - 62] | 38 | 38 | 0 | 38 |
| FPS014M | 32 [0 - 64] | 41 | 43 | -2 | 42 |
| FPS015M | 32 [0 - 64] | 42 | 39 | 3 | 40.5 |
| FPS016M | 31 [0 - 62] | 36 | 32 | 4 | 34 |
| FPS019M | 32 [0 - 64] | 42 | 37 | 5 | 39.5 |
| FPS022M | 32 [0 - 64] | 44 | 44 | 0 | 44 |
| FPS025M | 32 [0 - 64] | 39 | 40 | -1 | 39.5 |
| FPS026M | 31 [0 - 62] | 41 | 41 | 0 | 41 |
| FPS027M | 32 [0 - 64] | 45 | 45 | 0 | 45 |
| FPS028M | 32 [0 - 64] | 43 | 40 | 3 | 41.5 |
| FPS029M | 32 [0 - 64] | 43 | 44 | -1 | 43.5 |
| FPS030M | 32 [0 - 64] | 45 | 40 | 5 | 42.5 |
| Mean (SD) | | | | | 39.714 (3.129) |
| % Coefficient of Variation (CV) | | | | | 7.88 |
| P-value for Wilcoxon’s Paired Test | | | | | 0.057 |

* PGS range based on the number of genotyped SNPs available on the GSA for each family for PGS computation

**Supplementary Table 35**. PW-PGS [NumSNPs=39]

| FamID | Num SNPs available  [PGS range*] | PGS based on cord blood genotype | PGS based on prediction | Difference between cord blood and predicted PGS | Mean PGS –  Cord blood and predicted |
| --- | --- | --- | --- | --- | --- |
| FPS001M | 25 [0 - 50] | 37 | 38 | -1 | 37.5 |
| FPS002M | 24 [0 - 48] | 35 | 34 | 1 | 34.5 |
| FPS003M | 25 [0 - 50] | 32 | 34 | -2 | 33 |
| FPS005M | 25 [0 - 50] | 31 | 34 | -3 | 32.5 |
| FPS006M | 25 [0 - 50] | 37 | 38 | -1 | 37.5 |
| FPS008M | 23 [0 - 46] | 31 | 34 | -3 | 32.5 |
| FPS009M | 25 [0 - 50] | 36 | 35 | 1 | 35.5 |
| FPS011M | 25 [0 - 50] | 38 | 39 | -1 | 38.5 |
| FPS012M | 25 [0 - 50] | 34 | 33 | 1 | 33.5 |
| FPS013M | 24 [0 - 48] | 36 | 35 | 1 | 35.5 |
| FPS014M | 25 [0 - 50] | 40 | 38 | 2 | 39 |
| FPS015M | 25 [0 - 50] | 37 | 38 | -1 | 37.5 |
| FPS016M | 24 [0 - 48] | 34 | 32 | 2 | 33 |
| FPS019M | 25 [0 - 50] | 36 | 36 | 0 | 36 |
| FPS022M | 25 [0 - 50] | 37 | 35 | 2 | 36 |
| FPS025M | 25 [0 - 50] | 36 | 37 | -1 | 36.5 |
| FPS026M | 24 [0 - 48] | 35 | 36 | -1 | 35.5 |
| FPS027M | 25 [0 - 50] | 34 | 37 | -3 | 35.5 |
| FPS028M | 25 [0 - 50] | 40 | 35 | 5 | 37.5 |
| FPS029M | 24 [0 - 48] | 32 | 30 | 2 | 31 |
| FPS030M | 25 [0 - 50] | 31 | 35 | -4 | 33 |
| Mean (SD) | | | | | 35.286 (2.234) |
| % Coefficient of Variation (CV) | | | | | 6.33 |
| P-value for Wilcoxon Paired Test | | | | | 0.690 |

* PGS range based on the number of genotyped SNPs available on the GSA for each family for PGS computation

**Supplementary Table 36**. ADHD-PGS [NumSNPs = 27]

| FamID | Num SNPs available  [PGS range*] | PGS based on cord blood genotype | PGS based on prediction | Difference between cord blood and predicted PGS | Mean PGS –  Cord blood and predicted |
| --- | --- | --- | --- | --- | --- |
| FPS001M | 17 [0 - 34] | 18 | 21 | -3 | 19.5 |
| FPS002M | 18 [0 - 36] | 24 | 22 | 2 | 23 |
| FPS003M | 18 [0 - 36] | 17 | 21 | -4 | 19 |
| FPS005M | 18 [0 - 36] | 21 | 20 | 1 | 20.5 |
| FPS006M | 18 [0 - 36] | 22 | 22 | 0 | 22 |
| FPS008M | 17 [0 - 34] | 22 | 25 | -3 | 23.5 |
| FPS009M | 17 [0 - 34] | 18 | 16 | 2 | 17 |
| FPS011M | 17 [0 - 34] | 22 | 25 | -3 | 23.5 |
| FPS012M | 18 [0 - 36] | 19 | 19 | 0 | 19 |
| FPS013M | 17 [0 - 34] | 18 | 19 | -1 | 18.5 |
| FPS014M | 18 [0 - 36] | 21 | 21 | 0 | 21 |
| FPS015M | 17 [0 - 34] | 21 | 23 | -2 | 22 |
| FPS016M | 18 [0 - 36] | 16 | 18 | -2 | 17 |
| FPS019M | 18 [0 - 36] | 21 | 18 | 3 | 19.5 |
| FPS022M | 18 [0 - 36] | 16 | 15 | 1 | 15.5 |
| FPS025M | 16 [0 - 32] | 18 | 20 | -2 | 19 |
| FPS026M | 18 [0 - 36] | 21 | 21 | 0 | 21 |
| FPS027M | 17 [0 - 34] | 19 | 16 | 3 | 17.5 |
| FPS028M | 18 [0 - 36] | 25 | 21 | 4 | 23 |
| FPS029M | 17 [0 - 34] | 20 | 24 | -4 | 22 |
| FPS030M | 16 [0 - 32] | 16 | 14 | 2 | 15 |
| Mean (SD) | | | | | 19.905 (2.567) |
| % Coefficient of Variation (CV) | | | | | 12.90 |
| P-value for Wilcoxon’s Paired Test | | | | | 0.551 |

* PGS range based on the number of genotyped SNPs available on the GSA for each family for PGS computation

**Supplementary Table 37**. ChOB-PGS [NumSNPs=22]

| FamID | Num SNPs available  [PGS range*] | PGS based on cord blood genotype | PGS based on prediction | Difference between cord blood and predicted PGS | Mean PGS –  Cord blood and predicted |
| --- | --- | --- | --- | --- | --- |
| FPS001M | 13 [0 - 26] | 17 | 16 | 1 | 16.5 |
| FPS002M | 11 [0 - 22] | 10 | 12 | -2 | 11 |
| FPS003M | 13 [0 - 26] | 15 | 13 | 2 | 14 |
| FPS005M | 13 [0 - 26] | 15 | 13 | 2 | 14 |
| FPS006M | 13 [0 - 26] | 17 | 18 | -1 | 17.5 |
| FPS008M | 13 [0 - 26] | 18 | 14 | 4 | 16 |
| FPS009M | 13 [0 - 26] | 15 | 13 | 2 | 14 |
| FPS011M | 11 [0 - 22] | 15 | 15 | 0 | 15 |
| FPS012M | 13 [0 - 26] | 15 | 14 | 1 | 14.5 |
| FPS013M | 11 [0 - 22] | 13 | 14 | -1 | 13.5 |
| FPS014M | 13 [0 - 26] | 19 | 15 | 4 | 17 |
| FPS015M | 13 [0 - 26] | 10 | 11 | -1 | 10.5 |
| FPS016M | 12 [0 - 24] | 18 | 13 | 5 | 15.5 |
| FPS019M | 13 [0 - 26] | 17 | 19 | -2 | 18 |
| FPS022M | 13 [0 - 26] | 18 | 15 | 3 | 16.5 |
| FPS025M | 13 [0 - 26] | 15 | 15 | 0 | 15 |
| FPS026M | 12 [0 - 24] | 16 | 16 | 0 | 16 |
| FPS027M | 13 [0 - 26] | 17 | 19 | -2 | 18 |
| FPS028M | 11 [0 - 22] | 13 | 14 | -1 | 13.5 |
| FPS029M | 13 [0 - 26] | 16 | 11 | 5 | 13.5 |
| FPS030M | 12 [0 - 24] | 15 | 13 | 2 | 14 |
| Mean (SD) | | | | | 14.929 (2.027) |
| % Coefficient of Variation (CV) | | | | | 13.578 |
| P-value for Wilcoxon’s Paired Test | | | | | 0.071 |

* PGS range based on the number of genotyped SNPs available on the GSA for each family for PGS computation

**Supplementary Figure 1.** Mean and standard deviation of SNPclass distribution across the 21 study families in the study population, by individual SNP base-pairing **(A)** – SNP Base-pairing AC; **(B)** – SNP Base-pairing AG; **(C)** – SNP Base-pairing TC; **(D)** – SNP Base-pairing TG

**Supplementary Figure 2.** Graphical representation of discrepancies between PGS based on direct genotyping of cord blood vs predicted from parental samples (certainty method + 90% CI for maternal SNPs) Bland-Altman plots to illustrate the discrepancies between polygenic scores (PGS) - Directly genotyped based on cord blood samples vs Derived based on Certainty method + Confidence Interval (CI) method using the 90% CI. **(A)** - Birthweight 2019 (BW2019-PGS); **(B)** - Depression (DEP-PGS); **(C)** - Birthweight 2016 (BW2016-PGS); **(D)** – Placental Weight (PW-PGS); **(E)** – Attention Deficit/Hyperactivity Disorder (ADHD-PGS); **(F)** – Childhood Obesity (ChOB-PGS)

**Supplementary Figure 3.** Graphical representation of discrepancies between PGS based on direct genotyping of cord blood vs predicted from parental samples (certainty method + 99% CI for maternal SNPs) Bland-Altman plots to illustrate the discrepancies between polygenic scores (PGS) - Directly genotyped based on cord blood samples vs Derived based on Certainty method + Confidence Interval (CI) method using the 99% CI. **(A)** - Birthweight 2019 (BW2019-PGS); **(B)** - Depression (DEP-PGS); **(C)** - Birthweight 2016 (BW2016-PGS); **(D)** – Placental Weight (PW-PGS); **(E)** – Attention Deficit/Hyperactivity Disorder (ADHD-PGS); **(F)** – Childhood Obesity (ChOB-PGS)

**Supplementary Figure 4.** Scatterplot to illustrate the relationship between estimated fetal fraction and percent accuracy in SNPs predicted using the “CI” method (All Study Families). **(A)** – Overall Accuracy (All SNPs predicted with the “within CI” method; **(B)** – SNPs within Fetal SNP class F2; **(C)** - SNPs within Fetal SNP class F4; **(D)** – SNPs within Fetal SNP class F5; **(E)** – SNPs within Fetal SNP class F6; **(F)** – SNPs within Fetal SNP class F8. Data is presented for SNPs with base-pairing A/C

**Supplementary Figure 5.** Scatterplot to illustrate the relationship between estimated fetal fraction and percent accuracy in SNPs predicted using the “CI” method (FF Outlier Study Families excluded). **(A)** – Overall Accuracy (All SNPs predicted with the “within CI” method; **(B)** – SNPs within Fetal SNP class F2; **(C)** - SNPs within Fetal SNP class F4; **(D)** – SNPs within Fetal SNP class F5; **(E)** – SNPs within Fetal SNP class F6; **(F)** – SNPs within Fetal SNP class F8. Data is presented for SNPs with base-pairing A/C
